# Hypoxia-inducible CircPFKFB4 Promotes Breast Cancer Progression by Facilitating the CRL4^DDB2^ E3 Ubiquitin Ligase-mediated p27 Degradation

**DOI:** 10.7150/ijbs.72842

**Published:** 2022-06-06

**Authors:** Hang Chen, Rui Yang, Lei Xing, Bin Wang, Dawei Liu, Xiaoqiang Ou, Yumei Deng, Rong Jiang, Junxia Chen

**Affiliations:** 1 Department of Cell Biology and Genetics, Chongqing Medical University, 1 Yixueyuan Road, Chongqing 400016 P.R. China.; 2 Department of Endocrine and breast surgery, The First Affiliated Hospital of Chongqing Medical University, 1 Yixueyuan Road, Chongqing 400016, P.R. China.; 3 Department of Oncology, Daping Hospital of Army Medical University, 10 Changjiang Branch Road, Chongqing 400042, P.R. China.; 4 Department of anesthesiology, Yongchuan Hospital of Chongqing Medical University, 439 XuanHua Road, Chongqing 402160, P.R. China.; 5 Laboratory of Stem Cells and Tissue Engineering, Chongqing Medical University, 1 Yixueyuan Road, Chongqing 400016, P.R. China.

**Keywords:** Hypoxia, Breast cancer, circPFKFB4, CRL4^DDB2^ E3 ubiquitin ligase, p27

## Abstract

Hypoxic microenvironment and circular RNAs (circRNAs) have shown critical implications in breast cancer (BC) progression. However, the specific functions and underlying mechanisms of circRNAs in BC under hypoxia remain largely unknown. We first screened for differentially expressed circRNAs in normoxic and hypoxic MCF-7 cells using circRNA microarray. A novel hypoxia-induced circRNA, circPFKFB4, was identified. Clinical investigation showed that circPFKFB4 was highly expressed in BC tissues and cell lines, and its overexpression was positively correlated with the advanced clinical stage and poor prognosis of BC patients. Functionally, circPFKFB4 promoted the proliferation of BC cells both *in vitro* and *in vivo*. Mechanistically, HIF1α bound to hypoxia response elements in the promoter region of the PFKFB4 gene to facilitate the biogenesis of circPFKFB4 under hypoxia. Hypoxia-induced circPFKFB4 directly bound to both DDB1 and DDB2 and promoted the CRL4^DDB2^ E3 ubiquitin ligase assembly, resulting in p27 ubiquitination and BC progression under hypoxia. Our findings revealed a novel interaction between circPFKFB4 and the CRL4^DDB2^ E3 ubiquitin ligase, suggesting that circPFKFB4 might serve as a promising biomarker and therapeutic target for BC.

## Introduction

Breast cancer (BC) is the most common cancer in women and the leading cause of cancer-related deaths worldwide 1. BC is recognized as a heterogeneous disease. Targeted therapies based on traditional pathological characterization and immunohistochemistry (IHC) results have significantly improved the prognosis of BC patients 2. Further exploration of the molecular mechanisms of BC and discovery of the novel molecular biomarkers will provide more effective targeted therapies for BC.

The hypoxic microenvironment is a landmark characteristic of most solid tumors, which arises from the rapid proliferation of tumor cells, heteromorphic tumor structure, and aberrant structure and function of tumor vessels 3, 4. Hypoxia plays essential roles in angiogenesis, proliferation, metastasis, immune escape, and chem/radioresistance of BC 5, 6. Hypoxia-inducible factor 1α (HIF1α) is a central regulator in the hypoxia-induced microenvironment 7. Under hypoxia, HIF1α protein is stabilized and translocated to the nucleus where it binds to the hypoxia response elements (HREs) of genes, controlling the transcription of these genes and driving cancer cells to adapt to hypoxic stress 8, 9. Nevertheless, the molecular mechanism of BC progression under hypoxia remains to be elucidated.

Circular RNAs (circRNAs), a class of abundant and ubiquitous non-coding RNAs characterized by covalently closed continuous loops with the absence of both 5′ caps and 3′ poly (A) tails, are produced by precursor mRNA through back-splicing in eukaryotes 10, 11. Until now, substantial work has been done to characterize the expression of cell-specific and tissue-specific circRNAs in eukaryotes. CircRNAs can serve as microRNA (miRNA) sponges, RNA-binding protein (RBP) sponges, and templates for protein translation to regulate numerous biological processes, especially in cancer biology 12. In hypoxic environments, tumor cells generate various circRNAs with multiple functions to coordinate the process of tumors. For example, hypoxia-elevated circRNA_403658 is up-regulated in bladder cancer tissues and hypoxia-exposed bladder cancer cells, which accelerates bladder cancer progression by activation of lactate dehydrogenase A (LDHA) 13. Li et al. have demonstrated that circMAT2B promotes the malignant phenotype and glycolysis of hepatocellular carcinoma under hypoxia by up-regulating the expression of pyruvate kinase M2 (PKM2), the target gene of miR-338-3p 14. In addition, hypoxia-induced circELP3, which is strongly associated with advanced tumor stage and metastasis, promotes the malignant phenotype and cisplatin resistance of bladder cancer cells 15. However, the underlying functions and mechanisms of most circRNAs in human malignancies under hypoxic conditions have not been thoroughly clarified.

E3 ubiquitin ligases, a class of pivotal enzymes in the ubiquitin-proteasome proteolysis system, are essential for the regulation of biological processes such as cell cycle, division, and apoptosis. The Cullin-RING ubiquitin ligases (CRLs) comprise the largest family of E3 ligases in human 16. Generally, the CRLs include cullin, ring finger, adaptor, and substrate receptor proteins and target substrates for polyubiquitination through dedicated substrate receptors 17. For instance, the DDB1-DDB2-CUL4-RBX1 (CRL4^DDB2^) E3 ubiquitin ligase consists of damage-specific DNA-binding protein 1 (DDB1), damage-specific DNA-binding protein 2 (DDB2), cullin 4A (CUL4A), and RING-box protein 1 (RBX1), which exerts critical roles in various diseases such as cancers by mediating the ubiquitination and degradation of various proteins including histones H2A, H3, H4, progestin and adipoQ receptor 3 (PAQR3), and p27 18. Interestingly, the down-regulation of miR-34b-5p impairs the suppression of tumorigenicity 7 (ST7) stability mediated by CRL4^DCAF4^ E3 ligase, leading to the colitis-associated cancer tumorigenesis 19. However, the interactions between circRNAs and CRLs in BC under hypoxia have not been reported before.

In our study, we identified a novel hypoxia-induced circRNA circPFKFB4 (hsa_circ_0124008), which was significantly elevated in BC cells/tissues and was closely associated with advanced clinical stage and poor prognosis. HIF1α stimulated the generation of circPFKFB4 under hypoxia. Functional assays revealed that circPFKFB4 enhanced BC cells growth and tumorigenesis *in vitro* and* in vivo*. Mechanistically, circPFKFB4 increased the binding of DDB1 and DDB2, enhanced DDB2 stability, and further facilitated the assembly of the CRL4^DDB2^ ubiquitin ligase, thus eventually promoting the degradation of p27 and development of BC. Collectively, our findings revealed that circPFKFB4 participated in the response of BC cells to hypoxia and might serve as a promising therapeutic target and biomarker for BC.

## Materials and Methods

### Cell culture

BC cell lines (BT-474, MDA-MB-231, MDA-MB-453, and MCF-7) and human normal breast epithelial cell line (MCF-10A) were provided by Molecular Medicine and Cancer Research Center of Chongqing Medical University (China). The above cells were cultured in the cell medium supplemented with 10% fetal bovine serum (FBS, Gibco, Carlsbad, CA, USA) in a humidified incubator (5% CO_2_ at 37 °C). For hypoxic governance, the cells were cultured at 37°C in a hypoxic incubator containing 1% O_2_, 5% CO_2,_ and 94% N_2_.

### Clinical samples

100 pairs of BC tissues and corresponding paracancerous tissues were obtained from BC patients who underwent radical resection at the First Affiliated Hospital of Chongqing Medical University (China). All tissue samples were stored in liquid nitrogen until use. All tissues were diagnosed by two experienced pathologists independently. Ethical approval was identified by the Ethics Committee of the First Affiliated Hospital of Chongqing Medical University.

### Quantitative real-time polymerase chain reaction (qRT-PCR)

The total RNA was extracted using TRIzol reagent (Invitrogen, Carlsbad, CA, USA) and reverse-transcribed into complementary DNA (cDNA) using the PrimeScript RT Reagent Kit (Takara, Otsu, Japan) according to the recommended procedure. qRT-PCR was carried out using the Bio-Rad CFX96 system (Bio-Rad, Hercules, CA, USA) and SYBR Green Real-time PCR Master Mix Kit (Takara, Dalian, China). All transcript levels were calculated by using the comparative cycle threshold (ct) method. The primers used are provided in Additional file 1: Table S1.

### Microarray analysis

The total RNA was extracted using TRIzol reagent (Takara), evaluated using the Nanodrop 2000 (Thermo Fisher Scientific, Waltham, MA, USA) and Agilent Bioanalyzer 2100 (Agilent Technologies, PaloAlto, CA, USA), and then purified with rRNA depletion (Epicentre, Madison, WI, USA). The differentially expressed circRNAs and mRNAs were screened using the Agilent Human ceRNA MicroArray 2019 (Agilent Technologies). The differentially expressed mRNAs in hypoxia-treated MCF-7 cells after circPFKFB4 knockdown were analyzed using the Agilent SurePrint G3 Human Gene Expression v3 Microarray (Agilent Technologies). Data analysis was conducted by OE Biotechnology Co., Ltd., (Shanghai, China).

### Nucleocytoplasmic separation

Nuclear and cytoplasmic RNA fractions were collected from cells using the PARIS™ kit (Invitrogen) following the manufacturer's instructions. Subsequently, the above RNA was reverse-transcribed into cDNA using the PrimeScript RT Reagent Kit (Takara). Finally, the RNA expression level of gene was detected using the SYBR Green Real-time PCR Master Mix Kit (Takara).

### RNase R and actinomycin D treatments

2 μg RNA was incubated with 3 U/μg RNase R (Epicenter Technologies, Madison, WI, USA) at 37 °C for 45 min. For actinomycin D treatment, the cells were cultured in the conventional medium containing 3 ug/mL actinomycin D (Sigma-Aldrich, Steinheim, Germany) at the indicated time points. Subsequently, the RNA was extracted from the manipulated cells by the standard procedures. The aforementioned RNA levels were then measured using qRT-PCR.

### Transfection and infection

Linear circPFKFB4 was synthesized and inserted into pLC5-ciR vector (Geneseed, Guangzhou, China) to construct the circPFKFB4 overexpression plasmid (circPFKFB4). Small interfering RNAs (siRNAs) against circPFKFB4 (si-1 and si-2) and their respective control reagents were purchased from Geneseed. Human HIF1α and DDB2 cDNAs were subcloned into pcDNA3.1 vector (RiboBio, Guangzhou, China) to construct HIF1α and DDB2 overexpression plasmids, respectively. siRNAs for HIF1α (si#1 and si#2) and DDB2 (si-DDB2) were obtained from RiboBio. Full-length of human DDB1 cDNA and its truncation variants were constructed into pcDNA3.1-3 × Flag vector (RiboBio). The wide-type (WT) and truncated DDB2 cDNA sequences were amplified and subcloned into pcDNA3.1-3 × glutathione S-transferase (GST) vector (RiboBio). For luciferase reporter assay, human PFKFB4 promoter fragments with putative HREs and corresponding mutants were cloned into vector pGL3-Basic (Genecreate, Wuhan, China). Plasmids and siRNAs were transfected into BC cells using Lipofectamine 2000 (Invitrogen). The lentiviral vectors (pLC5-ciR-circPFKFB4, CMV-MCS-EF1-P2A-DDB2, U6-MCS-PGK-EGFP-si-1, and U6-CMV-P2A-BSD-si-DDB2, Hanbio, Shanghai, China) were co-transfected with packaged psPAX2 and pMD2.G plasmids into HEK293T cells. The viral supernatants were harvested 48 and 72 h after transfection and filtered through 0.22-mm filters (Millipore, Billerica, MA, USA). BC cells were subjected to lentivirus infection and then selected using puromycin (Hanbio) for 2 weeks to obtain cells stably expressing ideal genes.

### Cell Counting Kit-8 (CCK-8) assay

BC cells were plated into 96-well plates at 1.0 × 10^3^ cells/well in triplicate. The cells were incubated with CCK-8 reagent (Hanbio) at indicated times for 1.5 h. The optical density (OD) value of each well at the wavelength of 450 nm was measured using a microplate reader (Thermo Fisher Scientific).

### Colony formation assay

BC cells were plated into 6-well plates (1.0 × 10^3^ cells/well) and cultured for 2 weeks. The proliferating colonies were fixed with 4% paraformaldehyde and then stained with 0.1% crystal violet (Beyotime, Shanghai, China).

### 5-Ethynyl-20- deoxyuridine (EdU) assay

BC cells were seeded into 24-well plates and incubated with 100 mM EdU reagent for 2 h. Subsequently, the cells were fixed with 4% paraformaldehyde, permeabilized with 0.5% Triton X-100, and stained with corresponding reagents. Finally, the treated cells were imaged using a fluorescence microscope (Leica, Wetzlar, Germany).

### Cell cycle and apoptosis assays

The cells under different treatments were fixed with cold 70% ethanol at 4 °C for over 12 h and then dyed with a cell cycle analysis kit (Beyotime). For cell apoptotic analysis, the cells were harvested and stained with Annexin V-fluorescein isothiocyanate (FITC) and propidium iodide (PI). Finally, the cell cycle and apoptosis were evaluated using the flow cytometer FACS Calibur (Becon Dickinson, Franklin Lakes, NJ, USA). Hoechst 33342 and Tdt-mediated UTP nick-end labeling (TUNEL) stains (Beyotime) were utilized to detect cell apoptosis and observed under a fluorescent microscope (Leica).

### Luciferase reporter assay

BC cells in 24-well plates were co-transfected with the wild or mutant HREs reporter plasmids (Genecreate), together with HIF1α and renilla luciferase plasmids. 48 h later, the luciferase activity was measured using the Dual-Luciferase Reporter Assay Kit (Hanbio) and calculated as the ratio of firefly to renilla luciferase activity.

### Western blot analysis

The proteins extracted from cells were separated by sodium dodecyl sulfate-polyacrylamide gel electrophoresis (SDS-PAGE) and transferred onto polyvinylidene fluoride (PVDF) membranes (Millipore). The membranes were blocked with 5% skim milk at room temperature for 2 h, incubated with the corresponding primary antibodies at 4 °C for 14 h, followed by incubation with the appropriate secondary antibodies at room temperature for 1.5 h. Finally, the signals of the bands were visualized using the enhanced chemiluminescence (ECL) detection system (Millipore).

### RNA immunoprecipitation (RIP) assay

Anti-DDB1 (Santa Cruz, CA, USA), anti-DDB2 (Santa Cruz), or anti-IgG (CST, Beverly, MA, USA) was incubated with the magnetic beads at 4 °C for 4 h to synthesize antibody-coated beads. Subsequently, the cell lysates were incubated with corresponding beads and rotated at 4 °C for 14 h. Then the co-precipitated RNAs and proteins were extracted and purified using the RNA Immunoprecipitation Kit (Geneseed), followed by qRT-PCR and western blot analyses, respectively.

### Tissue microarray (TMA) and *in situ* hybridization (ISH)

TMA (Outdo Biotech, Shanghai, China) including 280 BC tissues and 162 normal tissues were deparaffinized in xylene, rehydrated in alcohol, digested with proteinase K, and hybridized with circPFKFB4 probe (Digoxin-5'-GCAGTTGGTCATGCACACTATTGAGGAATATTG-3'-Digoxin) (Geneseed), followed by incubation with anti-Digoxin (Roche, Basel, Switzerland). After 3,3'-diaminobenzidine (DAB) staining, the tissues were photographed. The distribution and staining intensity were evaluated as previously described 20.

### Chromatin immunoprecipitation (ChIP)

The cells were crosslinked with methanal, lysed in ChIP buffer containing protein inhibitor cocktail, and then sonicated using the VirTis Virsonic 100 Ultrasonic Homogenizer. Subsequently, the samples were resuspended and incubated with HIF1α (CST) or IgG (Millipore) antibodies at 4 °C for 14 h. The protein G magnetic beads and the above antibody-chromatin mixture were incubated at 4 °C for 2 h. Then, DNA was eluted from antibody/magnetic beads, purified, and subjected to qRT-PCR. The primers used in ChIP are provided in Additional file 1: Table S1.

### Biotinylated RNA pull-down assay and mass spectrometry

The biotin-labeled circPFKFB4 probe (5'-TGGGCAGTTGGTCATGCACACTATTGAGGAATATTGGAA-3'-Biotin) (Geneseed) was incubated with the magnetic beads at 25 °C for 2 h. The probe-coated beads were incubated with cell lysates at 4 °C overnight to generate the biotin-labeled RNA-protein complex. Subsequently, the samples were washed and analyzed using western blot and mass spectrometry (5600-plus, AB SCIEX, Framingham, MA, USA).

### Immunofluorescence (IF) and fluorescence *in situ* hybridization (FISH)

The cells on coverslips were fixed with 4% paraformaldehyde, permeabilized with 0.1% Triton-100, blocked with 5% bovine serum albumin, and incubated with anti-DDB1 (Santa Cruz) or anti-DDB2 (Santa Cruz) at 4 °C for 14 h, followed by incubation with FITC-conjugated secondary antibody at 37 °C for 2 h. Subsequently, the samples were hybridized with the specific probe for circPFKFB4 (5'-CY3-GTTGGTCATGCACACTATTGAGGAA-CY3-3') (Geneseed) at 4 °C for 14 h and then dyed with 4′,6-Diamidino-2-Phenylindole (DAPI) at room temperature for 2 h. After sealing, the samples were imaged using a fluorescence microscope (Leica).

### Co-immunoprecipitation (Co-IP)

Antibodies for DDB1 (Santa Cruz), DDB2 (Santa Cruz), or IgG (CST) were crosslinked with magnetic beads at 4 °C for 4 h. The cells were lysed using IP lysis reagent in PierceTM Classic Magnetic IP/Co-IP Kit (Thermo Fisher Scientific). The antibody-coated magnetic beads and cell lysates were mixed at 4 °C overnight. Proteins binding to specific antibodies were washed with buffer, dissolved in lane marker sample buffer, and subjected to western blot.

### Ubiquitination assay

The cells were transfected with indicated plasmids for 48 h, then processed with 20 μM MG132 (MedChemExpress, Monmouth Junction, NJ, USA) for 12 h, and lysed in NP-40 buffer (Beyotime), followed by sonication. The lysates were incubated with antibody-coated beads for 12 h at 4 °C. The ubiquitinated samples were washed and boiled for 10 min, followed by western blot analysis.

### Cycloheximide (CHX) chase assay

48 h after transefection, the cells treated with CHX (100 μg/mL, Genview, Tallahassee, FL, USA) at different time points were harvested and analyzed using western blot.

### Animal experiments

Female BALB/c nude mice (4-6 weeks) were purchased from Tengxin Biotechnology Co., Ltd (Chongqing, China) and maintained in a specific pathogen-free environment. For xenograft tumor experiments, 1 × 10^7^ MCF-7 cells in the logarithmic growth phase were resuspended in 50% matrigel with phenol red (BD Biosciences, Bedford, MA, USA) and subcutaneously injected into the mice (5 mice/group). Tumor volume was monitored every 7 days and calculated as length × width × width × 0.5. Survival analysis (10 mice/group) was performed on the mice with 70 days as a cutoff. After 28 days, the tumors were recorded using a small animal imaging system (Berthold, Wildbad, Germany) and the mice were euthanized. Finally, the excised tumors were weighted and measured using immunohistochemistry. All animal studies were identified by the Ethics Committee of the First Affiliated Hospital of Chongqing Medical University.

### Immunohistochemistry (IHC)

All fresh samples were fixed, hydrated, embedded, and sectioned. The sections were deparaffinized in xylene and rehydrated in alcohol. After antigen retrieval, the sections treated with hydrogen peroxide (H_2_O_2_) and goat serum were incubated with anti-DDB2 (Santa Cruz), anti-Ki67 (CST), and anti-p27 (CST) at 4 °C overnight. The aforementioned samples were incubated with secondary antibodies at 37 °C for 1 h. After diaminobenzidine and hematoxylin staining, these sections were dehydrated in alcohol and xylene and photographed under a microscope (Leica). The distribution and staining intensity were evaluated as previously described 20.

### Statistical analysis

Quantitative data are showed as the means ± standard deviation (SD). Differences among groups were assessed by the Student's t test or one-way analysis of variance (ANOVA), as appropriate. The chi-square test was used for correlation analysis between groups. Kaplan-Meier's method was used to analyze the survival rate. The univariate and multivariate Cox proportional hazards regression models were applied to estimate independent prognostic factors. The receiver operating characteristic (ROC) curve was employed to identify the diagnostic value of circPFKFB4. Statistical analyses were carried out using GraphPad Prism 7.0 (San Diego, CA, USA) and SPSS 22.0 (IBM, SPSS, Chicago, IL, USA). *P* < 0.05 was indicated of statistical significance.

## Results

### Identification and characterization of circPFKFB4 in BC cells under hypoxia

**Figure 1:**
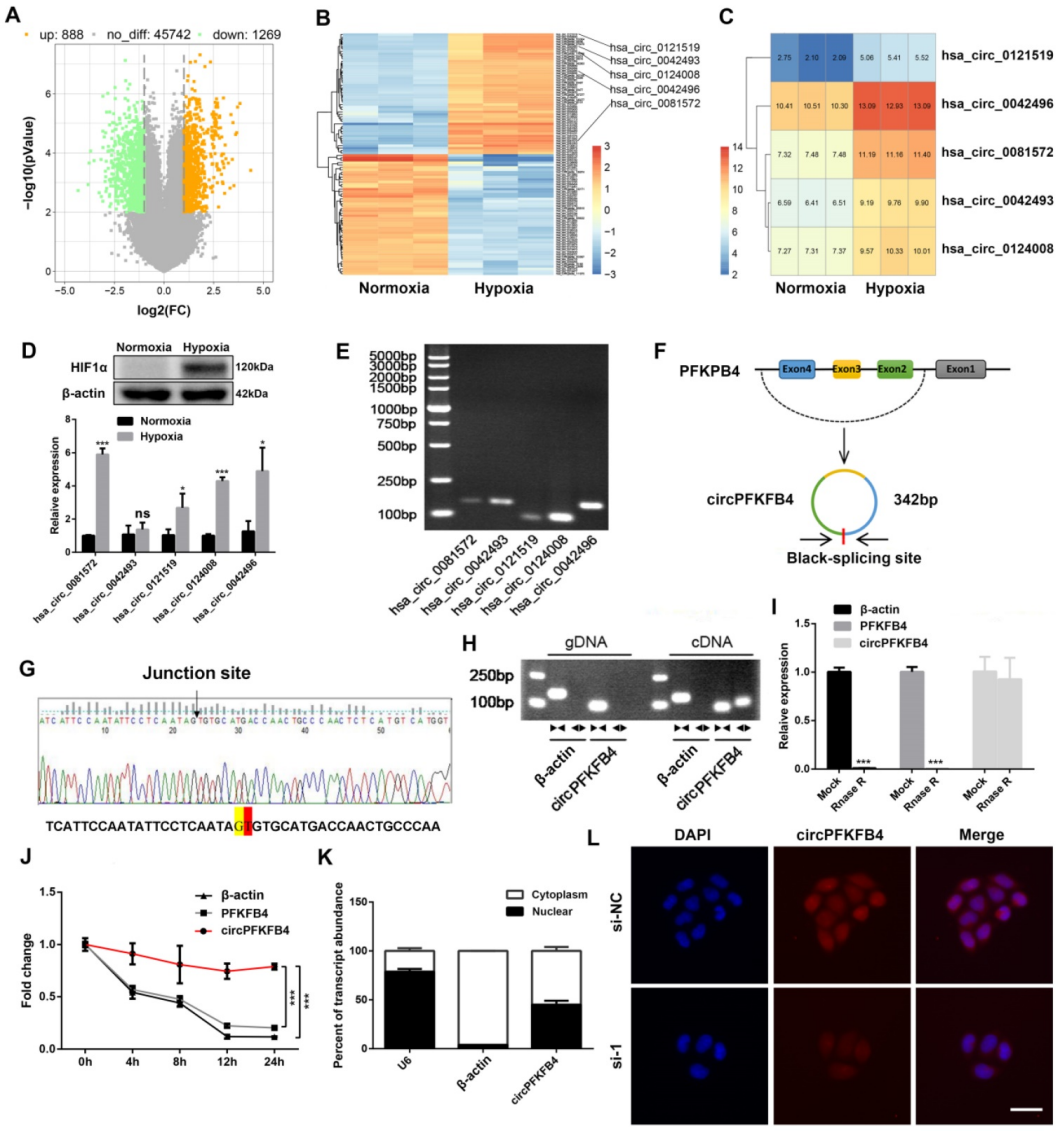
** CircPFKFB4 is validated and characterized in BC cells under hypoxia. (A)** Volcano plot showed the differentially expressed circRNAs in MCF-7 cells exposed to hypoxia (1% O_2_) for 48 h, as compared to normoxic MCF-7 cells. The orange and green plots indicate the statistically significant up-regulated and down-regulated circRNAs, respectively. **(B)** Heatmap showed the top 50 up-regulated and down-regulated circRNAs in hypoxic MCF-7 cells compared with normoxic MCF-7 cells. High and low expression levels are denoted in orange and blue, respectively. **(C)** Five up-regulated circRNAs in hypoxic MCF-7 cells were shown. **(D)** The expression of HIF1α in normoxic and hypoxic MCF-7 cells was monitored by western blot (upper panel). The expressions of hsa_circ_0081572, hsa_circ_0042493, hsa_circ_0121519, hsa_circ_0124008, and hsa_circ_042496 in MCF-7 cells after normoxia or hypoxia treatment for 48 h (lower panel) were analyzed using qRT-PCR. **(E)** RT-PCR combined with agarose electrophoresis observed the expressions of the above five circRNAs in MCF-7 cells under 48 h of hypoxia treatment. **(F)** Schematic diagram showed that circPFKFB4 was produced by exons 2, 3, and 4 of PFKFB4 gene. **(G)** Sanger sequencing confirmed the junction point of circPFKFB4. **(H)** The existence of circPFKFB4 and PFKFB4 in cDNA and gDNA from MCF-7 cells was validated using RT-PCR with convergent and divergent primers. **(I)** The RNA expression levels of β-actin, PFKFB4, and circPFKFB4 in RNA samples after treatment with RNase R were analyzed using qRT-PCR. **(J)** The relative RNA levels of β-actin, PFKFB4, and circPFKFB4 in hypoxic MCF-7 cells treated with actinomycin D for diverse periods of time were determined using qRT-PCR. **(K)** The expression of circPFKFB4 in the nuclear and cytoplasmic fractions of hypoxic MCF-7 cells was detected using qRT-PCR. U6 and β-actin served as nuclear and cytoplasm controls, respectively. **(L)** FISH assay for circPFKFB4 in MCF-7 cells cultured under hypoxia. Scale bar, 50 µm. Data are presented as mean ± SD and representative of three independent experiments in (D, I, J and K). *P<0.05, ***P<0.001, ns, no significance.

To investigate the involvement of hypoxia-induced circRNAs in the progression of BC under hypoxia, we performed a circRNA microarray analysis on normoxia- and hypoxia-disposed MCF-7 cells. We screened out 2157 differentially expressed circRNAs (fold change ≥ 2.0 and *P* value < 0.01) in hypoxia-treated MCF-7 cells, of which 888 were up-regulated and 1269 were down-regulated (Fig. 1A). Of note, only 5 of the top 50 up-regulated circRNAs could be identified with divergent primers because of the special structure of circRNAs (Fig. 1B and C). Next, the differential expression patterns of these five candidate circRNAs in normoxia- and hypoxia-treated MCF-7 cells and their expression levels in hypoxic MCF-7 cells were verified by qRT-PCR and RT-PCR combined with agarose electrophoresis, respectively. Among them, hsa_circ_0124008 showed the most significant expression and was increased in hypoxic MCF-7 cells (Fig. 1D and E). The genomic structure in the circBase database (http://www.circbase.org/) revealed that circPFKFB4 (hsa_circ_0124008) was derived from the back-splicing of exons 2, 3, and 4 of PFKFB4 gene (342 bp in total) (Fig. 1F). The circPFKFB4 back-splicing junction was validated by Sanger sequencing (Fig. 1G). Then, circPFKFB4 and linear PFKFB4 were amplified by divergent primers and convergent primers in genomic DNA (gDNA) and cDNA, respectively. As shown in Fig. 1H, circPFKFB4 could be amplify by the divergent primers in cDNA but not in gDNA, while linear PFKFB4 could not be amplify by the divergent primers in gDNA and cDNA. Then, the stability of circPFKFB4 in MCF-7 cells was confirmed by RNase R digestion and actinomycin D experiments. The results showed that the relative expressions of β-actin and PFKFB4 mRNA were significantly decreased, while circPFKFB4 was not rapidly degraded after treatments with RNase R and actinomycin D, illustrating the circular RNA structure of circPFKFB4 (Fig. 1I and J). In addition, cytoplasmic/nuclear fractionation and FISH assays suggested that circPFKFB4 presented in both the nucleus and cytoplasm of hypoxic BC cells (Fig. 1K, Fig. 1L and Fig. S1). In brief, circPFKFB4, an abundantly expressed and stable circRNA associated with hypoxia, might be involved in the development of BC under hypoxia.

### HIF1α elevates circPFKFB4 expression under hypoxia

**Figure 2:**
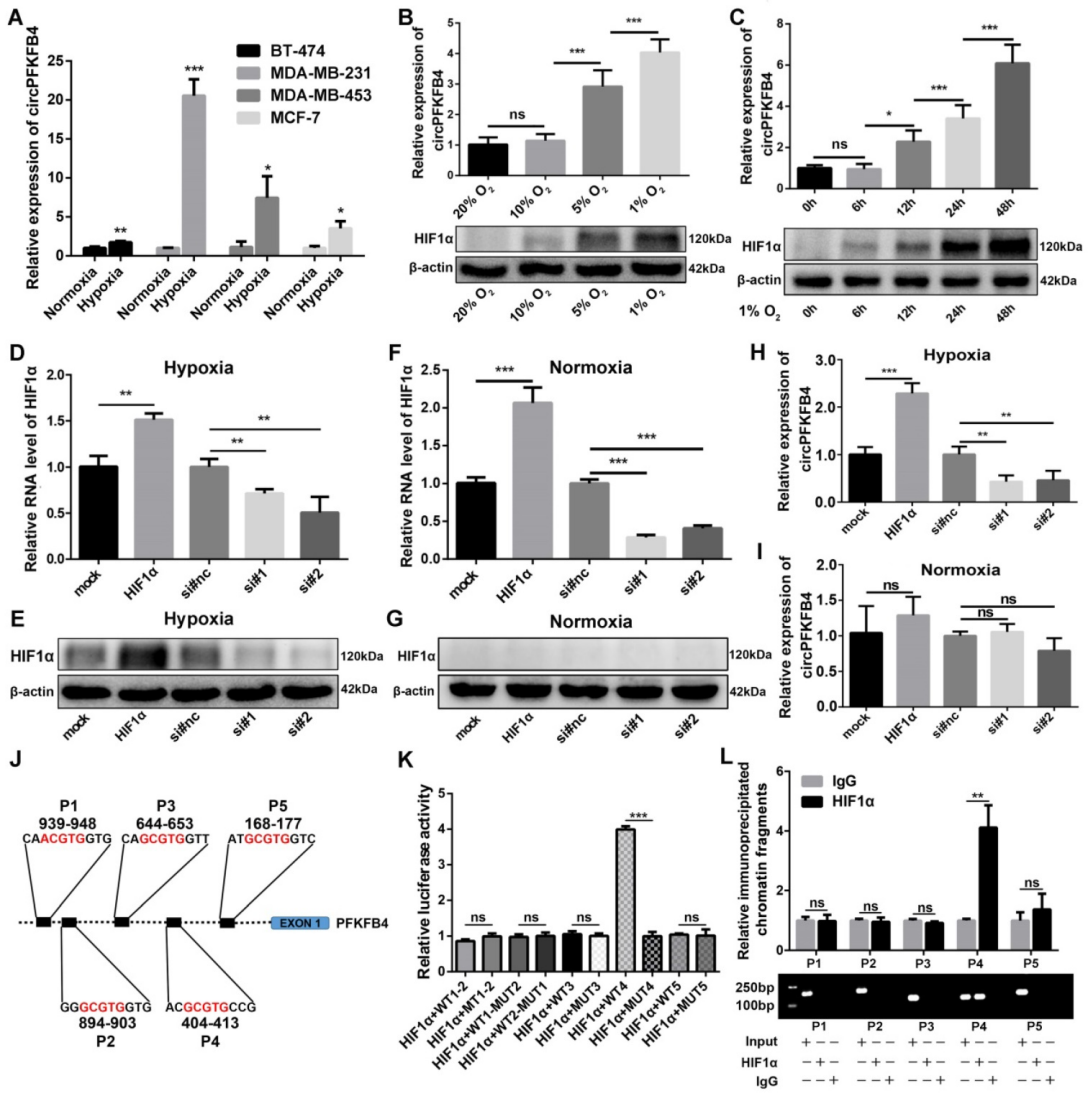
** The expression of circPFKFB4 is induced by HIF1α under hypoxia. (A)** qRT-PCR validation of circPFKFB4 expression in BC cell lines after normoxia or hypoxia treatment (1% O_2_, 48 h). **(B)** HIF1α protein level and RNA expression of circPFKFB4 in MCF-7 cells cultured under different concentrations of O_2_ for 1 day were analyzed using western blot and qRT-PCR, respectively. **(C)** CircPFKFB4 expression and HIF1α protein level in MCF-7 cells exposed to hypoxia (O_2_, 1%) for appointed times were detected using qRT-PCR and western blot, respectively. **(D-G)** The mRNA and protein levels of HIF1α in hypoxia- (D and E) and normoxia-treated (F and G) MCF-7 cells transfected with HIF1α overexpression plasmid (HIF1α) or siRNA (si#1 and si#2) were detected by qRT-PCR and western blot, respectively. **(H and I)** qRT-PCR verification of the relative circPFKFB4 RNA level in MCF-7 cells after up-regulation or down-regulation of HIF1α under hypoxia (H) and normoxia (I). **(J)** Schematic image of the potential HREs in PFKFB4 promoter region that could bind to HIF1α. **(K)** The relative luciferase activities were measured using luciferase reporter assay in hypoxic MCF-7 cells co-transfected with indicated luciferase reporter plasmids and ectopic plasmid of HIF1α. **(L)** ChIP followed by qPCR (upper panel) and PCR (lower panel) assays indicated that HIF1α was enriched at the PFKFB4 promoter in MCF-7 cells under hypoxia. Data are presented as mean ± SD and representative of three independent experiments in (A-D, F, H, I, K and L). *P<0.05, **P<0.01, ***P<0.001, ns, no significance.

To further verify the relationship between circPFKFB4 and hypoxia and the generation mechanism of circPFKFB4 under hypoxia, we detected the expression of circPFKFB4 in BC cell lines (BT-474, MDA-MB-231, MDA-MB-453, and MCF-7) with or without hypoxic management. CircPFKFB4 expression was up-regulated in hypoxia-cultured BC cells compared with BC cells cultured under normoxia (Fig. 2A). Next, MCF-7 cells were exposed to 20%, 10%, 5%, and 1% oxygen for 24 h, and then, HIF1α protein level and circPFKFB4 RNA level were detected. We found that the lower the oxygen concentration, the higher the HIF1α protein level and circPFKFB4 RNA level were detected (Fig. 2B). In addition, we detected the expressions of HIF1α and circPFKFB4 in MCF-7 cells cultured in an incubator with 1% oxygen concentration for 0, 6, 12, 24, and 48 h. The results showed that the longer the hypoxia treatment, the higher the expression of circPFKFB4 was observed (Fig. 2C). Considering that the transcription factor HIF1α can regulate the transcription of numerous genes under hypoxic conditions, including PFKFB4 (the parental gene of circPFKFB4). Hence, we hypothesized that the up-regulation of circPFKFB4 under hypoxia might be induced by HIF1α. We overexpressed and knocked down the expression of HIF1α under hypoxia and normoxia (Fig. 2D-G). As shown in Fig. 2H and I, HIF1α could affect the expression of circPFKFB4 under hypoxia rather than normoxia, indicating that circPFKFB4 was activated by hypoxia in a HIF1α-dependent manner. JASPAR database (http://jaspar.genereg.net/) indicated that five putative HREs were located in the promoter region of the PFKFB4 gene from 2 kb upstream of exon 1 (Fig. 2J). Dual-luciferase reporter assay illustrated that HIF1α enhanced the luciferase activity of the luciferase reporter vector carrying the wild-type promoter 4 (WT4) rather than the others under hypoxic conditions (Fig. 2K and Fig. S2A). Consistent with the results of the dual-luciferase reporter assay, ChIP assay demonstrated that the protein/DNA complexes containing P4 of the PFKFB4 promoter were precipitated with antibody against HIF1α under hypoxia (Fig. 2L). These results suggested that HIF1α elevated the level of circPFKFB4 by directly binding to the HRE 4 of the PFKFB4 promoter under hypoxia.

### CircPFKFB4 is up-regulated in BC cells and tissues and associated with clinicopathological parameters

**Figure 3:**
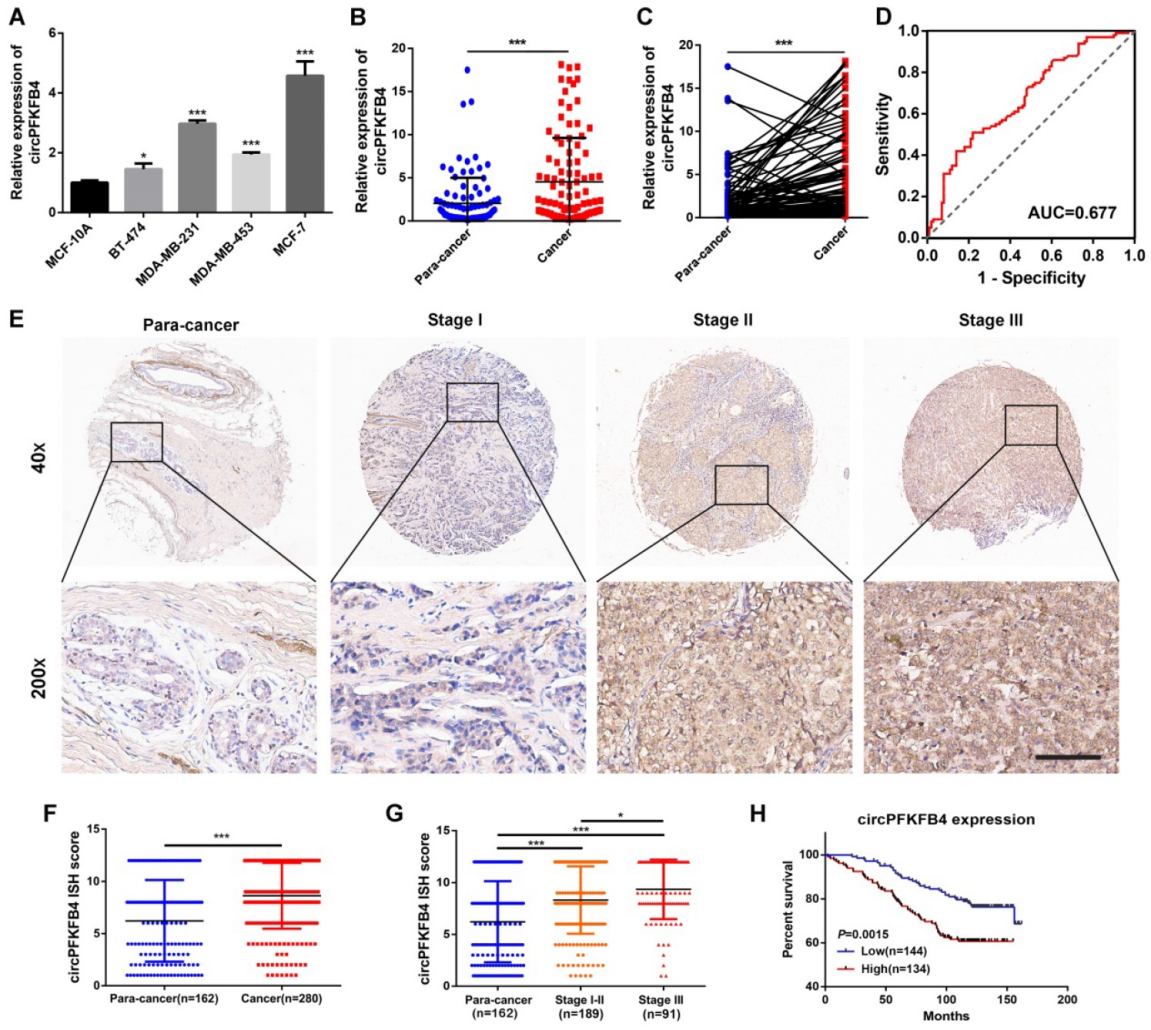
** CircPFKFB4 is highly expressed in BC and correlated with poor prognosis of BC patients. (A)** qRT-PCR analysis of circPFKFB4 in BC cell lines and MCF-10A cells. **(B and C)** The relative expression of circPFKFB4 in 100 pairs of BC tissues and adjacent normal tissues. **(D)** ROC analysis of circPFKFB4 in BC patients. **(E)** Representative images of circPFKFB4 expression in TMA were monitored by ISH. Scale bar, 100 µm. **(F and G)** The ISH scores for circPFKFB4 in TMA were quantified. **(H)** BC patients were dichotomized into two groups according to the median expression of circPFKFB4. Kaplan-Meier survival analysis of circPFKFB4 in BC patients. Data are presented as mean ± SD and representative of three independent experiments in (A). *P<0.05, **P<0.01, ***P<0.001.

To evaluate the clinical value of circPFKFB4 in BC, we detected the expression level of circPFKFB4 in BC cell lines and tissues. Higher circPFKFB4 expression was observed in four BC cell lines relative to MCF-10A cells (Fig. 3A). Moreover, compared with the paired normal breast tissues, circPFKFB4 expression was significantly increased in BC tissues (Fig. 3B and C). ROC analysis revealed that the area under the curve (AUC) was 0.677, hinting that circPFKFB4 was significant for the screening of BC tissues and non-cancerous tissues (Fig. 3D). The correlation analysis between circPFKFB4 expression and BC clinical characteristics revealed that higher circPFKFB4 expression was indicative of larger tumor size (Table 1). Subsequently, we further evaluated circPFKFB4 expression and its clinical value in BC using human TMA with 280 BC tissues and 162 paracancerous tissues. As shown in Fig. 3E-G and Table 2, the expression of circPFKFB4 was markedly increased in BC tissues and positively correlated with tumor size and tumor node metastasis (TNM) stage. Kaplan-Meier survival analysis showed that patients with higher circPFKFB4 expression exhibited a lower overall survival (Fig. 3H). Next, Cox proportional hazard analysis confirmed that circPFKFB4 could be regarded as an independent predictor of poor prognosis in BC patients (Table 3). In brief, circPFKFB4 was significantly up-regulated in BC cells and tissues and might be used as a reliable predictor for BC diagnosis and prognosis.

### CircPFKFB4 promotes hypoxic BC cells proliferation *in vitro* and *in vivo*

**Figure 4:**
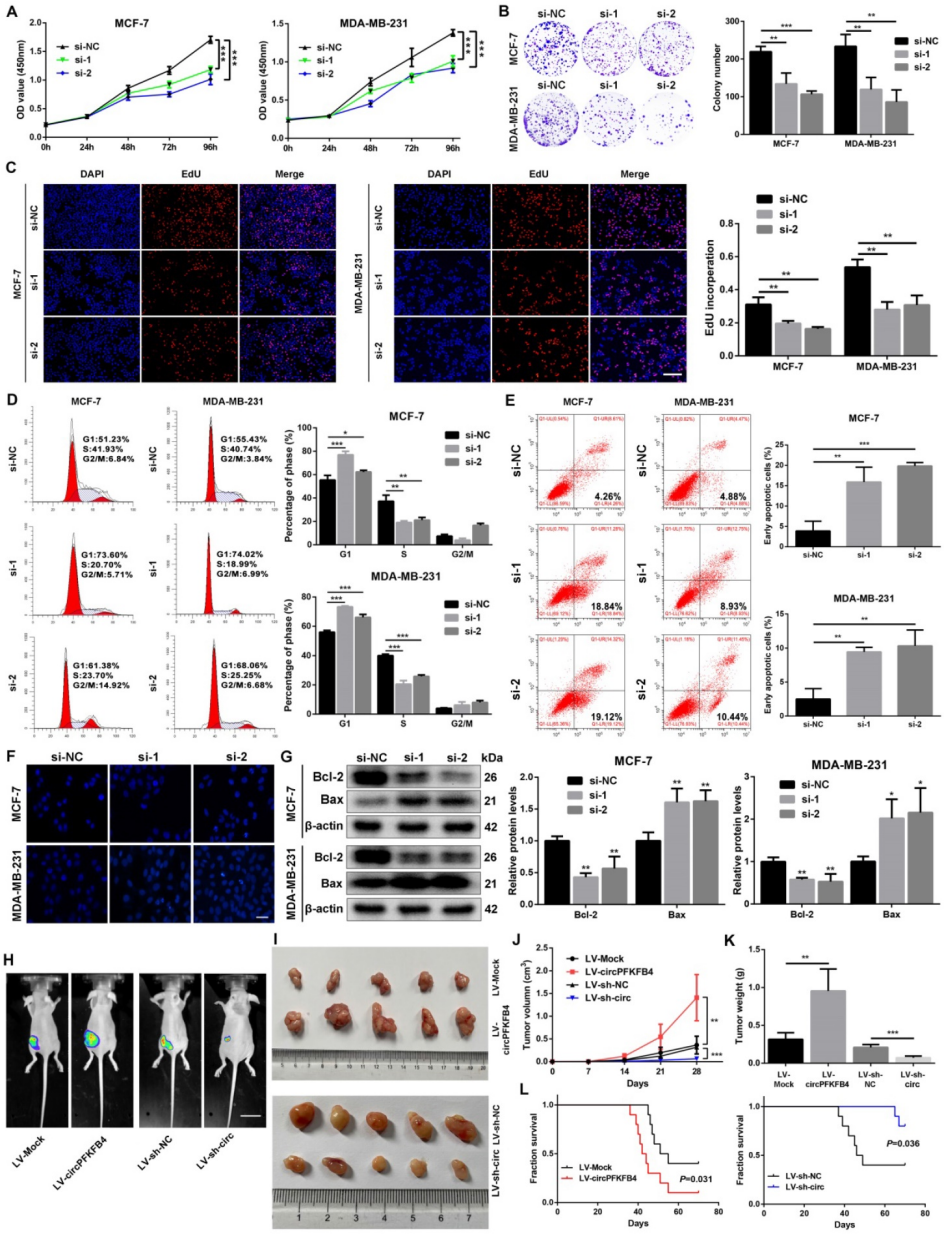
** CircPFKFB4 increases the proliferation of BC cells *in vitro* and *in vivo*. (A-C)** CCK-8 (A), colony formation (B), and EdU (C, scale bar, 200 µm) assays were performed to evaluate cell viability under hypoxia. **(D and E)** Flow cytometry was applied to measure cell cycle (D) and apoptosis rate (E) of hypoxic BC cells. **(F)** Hoechst 33342 staining indicated the morphological features of apoptosis in BC cells after knockdown of circPFKFB4 (si-1 and si-2) under hypoxic conditions. Scale bar, 50 µm. **(G)** The levels of apoptosis-related proteins under hypoxic circumstances were examined by western blot. **(H and I)** Representative images of bioluminescence (H, scale bar, 2 cm) and xenograft tumors (I, n = 5) in each group. **(J)** Tumor volumes were calculated every 7 days. The tumor growth curves were drawn and shown (n = 5). **(K)** Tumor weight (n = 5). **(L)** Kaplan-Meier survival curves of nude mice with different circPFKFB4 expressions (n=10). Data are presented as mean ± SD and representative of three independent experiments in (A-E and G). *P<0.05, **P<0.01, ***P<0.001, ns, no significance.

For the exploration of the biological functions of circPFKFB4 in hypoxic BC cells, subsequent functional and mechanistic experiments *in vitro* were performed under hypoxia. Firstly, overexpression plasmid and siRNAs for circPFKFB4 were synthesized and then transfected into BC cells. qRT-PCR results unveiled that overexpression plasmid or siRNAs for circPFKFB4 led to an increase or decrease in circPFKFB4 expression, but had no effect on the expression of PFKFB4 under hypoxia (Fig. S1). The results of CCK-8, colony formation, and EdU assays revealed that down-regulation of circPFKFB4 significantly decreased the proliferation of hypoxia-treated BC cells, while the opposite trend was found in BC cells transfected with circPFKFB4 overexpression plasmid (Fig. 4A-C and Fig. S3A-C). In addition, knocking down circPFKFB4 resulted in a significantly higher percentage of hypoxic BC cells in the G0/G1 phase and a lower percentage of cells in the S phase compared to si-NC treatment (Fig. 4D). Flow cytometry apoptosis assay demonstrated that depletion of circPFKFB4 led to an increase in the percentage of apoptotic BC cells under hypoxic conditions (Fig. 4E). Moreover, the apoptosis of hypoxic BC cells after knockdown of circPFKFB4 was significantly increased using Hoechst33342 and TUNEL staining (Fig. 4F and Fig. S3D). We also detected apoptosis-related proteins and found that Bcl-2 was significantly decreased, whereas Bax was markedly increased in hypoxic BC cells transfected with si-circPFKFB4 (Fig. 4G).

To further investigate the role of circPFKFB4 *in vivo*, MCF-7 cells with stably forced circPFKFB4 expression (LV-circPFKFB4) and knockdown circPFKFB4 expression (LV-sh-circ) were generated and confirmed using qRT-PCR (Fig. S4A and B). Xenograft tumor model assay unveiled that compared with the controls, overexpression of circPFKFB4 strikingly augmented the volume, weight, and growth rate of tumors, while knockdown of circPFKFB4 led to opposite results (Fig. 4H-K). Furthermore, IHC analysis revealed that circPFKFB4-overexpressing group exhibited higher levels of DDB2 and Ki67 as well as lower expression of p27, whereas circPFKFB4 silencing had the opposite effects (Fig. S4C). Kaplan-Meier analysis verified that the survival time of the nude mice in the LV-circPFKFB4 group was lower than those in the LV-Mock group, whereas the opposite results were obtained in the LV-sh-circ group (Fig. 4L). Together, these data illustrated that circPFKFB4 accelerated the progress of BC under hypoxia.

### CircPFKFB4 directly binds to both DDB1 and DDB2 and facilitates the assembly of the CRL4^DDB2^ ubiquitin ligase under hypoxia

**Figure 5:**
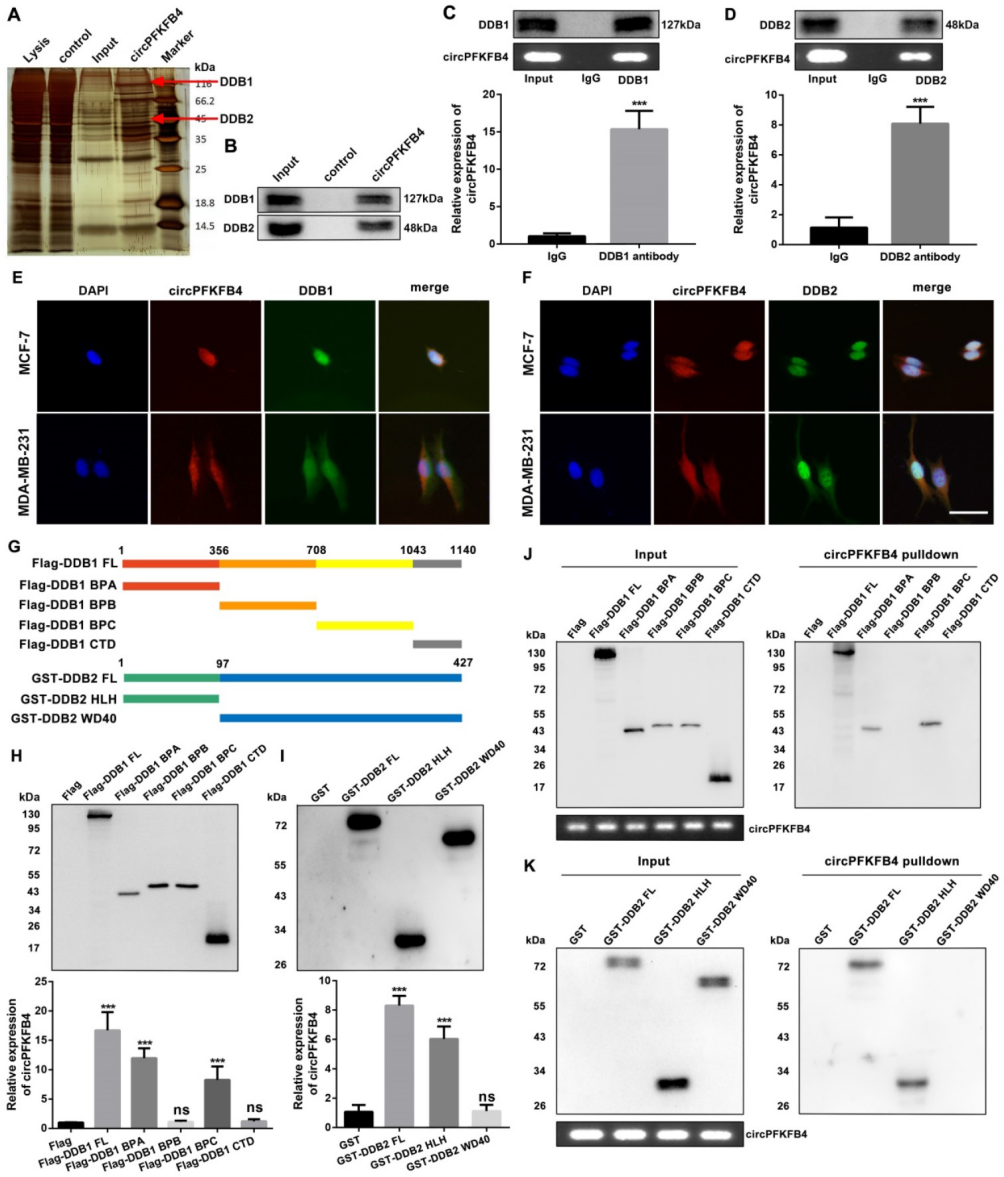
** CircPFKFB4 binds directly to both DDB1 and DDB2 under hypoxic conditions. (A and B)** CircPFKFB4-binding proteins were silver stained (A) and identified by mass spectrometry and western blot (B). **(C and D)** RIP assay was performed in cell lysates using anti-DDB1 (C), anti-DDB2 (D), or anti-IgG, followed by qRT-PCR and RT-PCR validation of circPFKFB4. **(E and F)** The localization of circPFKFB4 (red), DDB1 (E, green), and DDB2 (F, green) in hypoxic MCF-7 and MDA-MB-231 cells was observed using FISH-IF assay. Scale bar, 50 µm. **(G)** Schematic representation of DDB1 (upper panel) and DDB2 (lower panel)-truncated fragments. **(H)** MCF-7 cells were transfected with DDB1-truncated plasmids with 3 × Flag under hypoxia and then subjected to RIP assay. Proteins with 3 × Flag (upper panel) and circPFKFB4 (lower panel) were detected using western blot and qRT-PCR, respectively. **(I)** MCF-7 cells were transfected with DDB2-truncated plasmids with GST under hypoxia, followed by RIP assay. GST-tagged proteins (upper panel) and circPFKFB4 (lower panel) were detected by western blot and qRT-PCR, respectively. **(J and K)** Different domains of DDB1 (J) and DDB2 (K) truncations were pulled down by biotin-labeled probe of circPFKFB4 in hypoxia-cultured MCF-7 cells, followed using western blot. Data are presented as mean ± SD and representative of three independent experiments in (C, D, H and I). ***P<0.001, ns, no significance.

**Figure 6:**
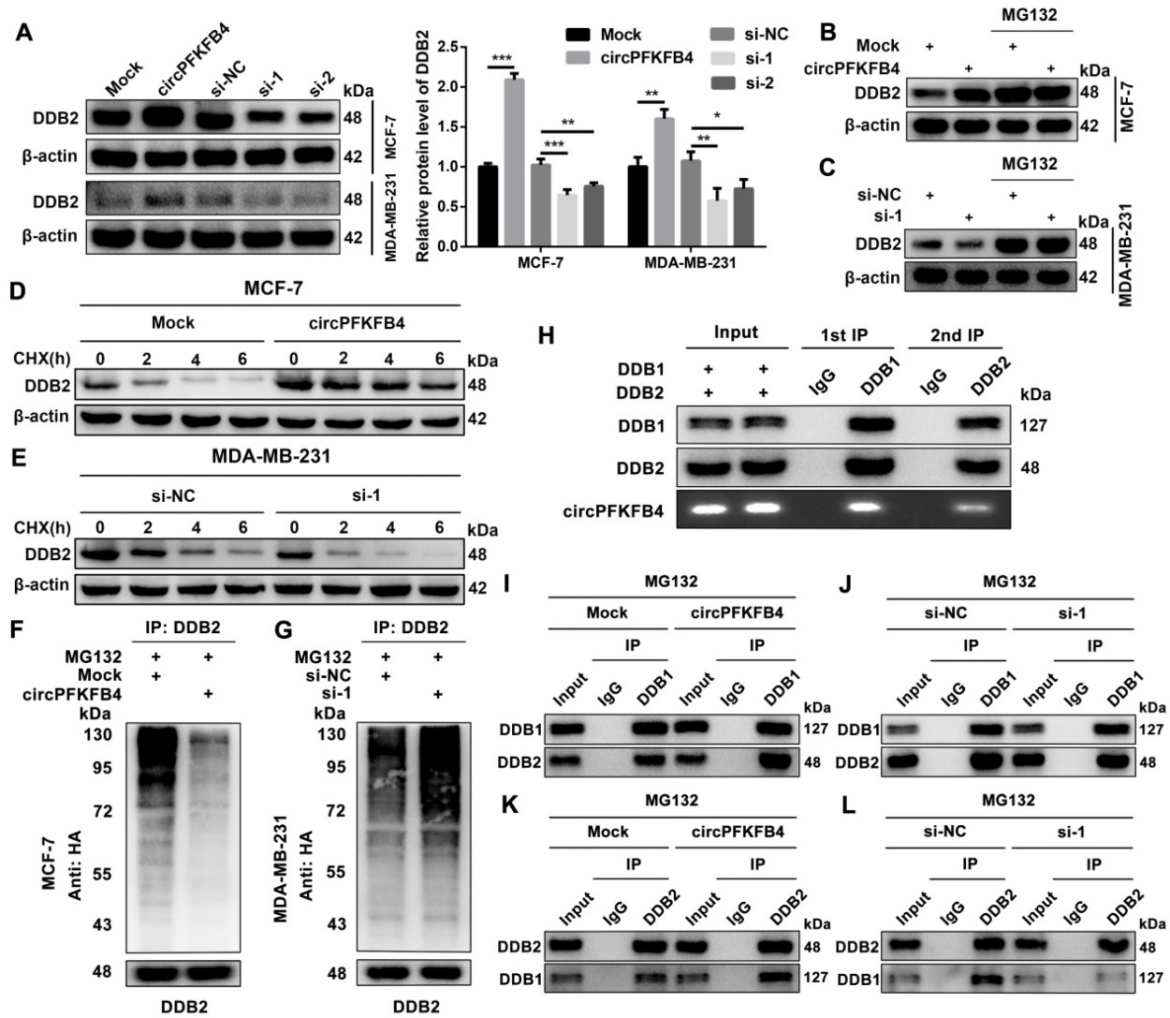
** CircPFKFB4 facilitates the assembly of CRL4^DDB2^ ubiquitin ligase under hypoxic circumstances. (A)** Western blot analysis of DDB2 protein levels in hypoxic MCF-7 and MDA-MB-231 cells after up-regulation or down-regulation of circPFKFB4. **(B and C)** MCF-7 (B) and MDA-MB-231 (C) cells bearing indicated vectors were processed with/without 20 μM MG132 (12 h) under hypoxia. DDB2 expression was detected by western blot. **(D and E)** The relative protein level of DDB2 in hypoxic MCF-7 (D) and MDA-MB-231 (E) cells treated with CHX at indicated times was measured using western blot analysis. **(F and G)** The ubiquitination level of DDB2 in hypoxic MCF-7 (F) and MDA-MB-231 (G) cells after circPFKFB4 up-regulation or down-regulation was analyzed by IP and western blot. **(H)** Two-step IP combined with western blot displayed the binding among circPFKFB4, DDB1, and DDB2 under hypoxia. **(I-L)** Co-IP assay was applied in hypoxic MCF-7 cell lysates using anti-DDB1, anti-DDB2, or anti-IgG, and then, the levels of DDB1 and DDB2 were detected by western blot. Data are presented as mean ± SD and representative of three independent experiments in (A). *P<0.05, **P<0.01, ***P<0.001.

To investigate the molecular mechanism of circPFKFB4 in promoting BC progression under hypoxia, we implemented biotin-labeled RNA pull-down assay combined with LC/MS analysis to explore the potential circPFKFB4-binding proteins and revealed that DDB1 and DDB2 were captured by the biotin-labeled probe against circPFKFB4 (Fig. 5A, Fig. 5B, Fig. S5 and Fig. S6). In addition, RIP analysis further displayed that circPFKFB4 directly bound to both DDB1 and DDB2 under hypoxic conditions (Fig. 5C and D). Subsequently, FISH-IF analysis revealed the co-localization of circPFKFB4 with DDB1 and DDB2 in hypoxic BC cells (Fig. 5E and F). The aforementioned results prompted us to explore that the domains of DDB1 and DDB2 were responsible for interaction with circPFKFB4 under hypoxia. Domain mapping assay combined with RIP demonstrated that under hypoxic conditions, circPFKFB4 bound to the WD repeat β-propeller A (BPA, aa 1 to 356) and WD repeat β-propeller C (BPC, aa 708 to 1043) domains of DDB1, and bound with an N-terminal helix-loophelix segment (HLH, aa 1 to 97) of DDB2 (Fig. 5G-I). RNA pull-down assay showed that the BPA and BPC domains of DDB1 were enriched on the complex precipitated by circPFKFB4 probe, while circPFKFB4 directly interacted with the HLH domain of DDB2 under hypoxia (Fig. 5J and K). Previous studies have indicated that circRNAs can affect the assembly and stability of enzymes 21. Therefore, we checked the effect of circPFKFB4 on each component of the CRL4^DDB2^ ubiquitin ligase and found that circPFKFB4 increased the protein level of DDB2 instead of DDB1, CUL4A, or RBX1 under hypoxic conditions (Fig. 6A, Fig. S7A and Fig. S7B). Furthermore, the regulation of DDB2 mRNA level by circPFKFB4 was not observed in hypoxic BC cells (Fig. S7C and D). Considering that DDB2 can be ubiquitinated, we next examined the effect of circPFKFB4 on DDB2 ubiquitination and proteasomal degradation. After the addition of proteasome inhibitor (MG132), the up-regulation of DDB2 protein level caused by ectopic circPFKFB4 expression was significantly restored under hypoxia (Fig. 6B). Furthermore, MG132 effectively stabilized the expression of DDB2 following depletion of circPFKFB4 (Fig. 6C). CHX chase assay displayed that the up-regulation of circPFKFB4 markedly extended the half-life of DDB2, while circPFKFB4 silencing produced the opposite effect under hypoxic conditions (Fig. 6D and E). In addition, the level of ubiquitylated DDB2 was decreased in cells transfected with circPFKFB4 overexpression plasmid but increased in cells treated with circPFKFB4 siRNA, suggesting that circPFKFB4 prevented the ubiquitin-proteasomal degradation of DDB2 in hypoxic BC cells (Fig. 6F and G). Subsequently, the results of two-step immunoprecipitation demonstrated that circPFKFB4, DDB1, and DDB2 formed a trimer, suggesting that circPFKFB4 was closely bound to the CRL4^DDB2^ ubiquitin ligase under hypoxia (Fig. 6H). Co-IP experiment revealed that circPFKFB4 enhanced the association between DDB1 and DDB2 in hypoxic BC cells (Fig. 6I-L). Collectively, these results suggested that circPFKFB4 might suppress DDB2 degradation and further facilitate the assembly of CRL4^DDB2^ ubiquitin ligase under hypoxic conditions.

### CircPFKFB4 increases the degradation of p27 via the CRL4^DDB2^ ubiquitin ligase under hypoxia

**Figure 7:**
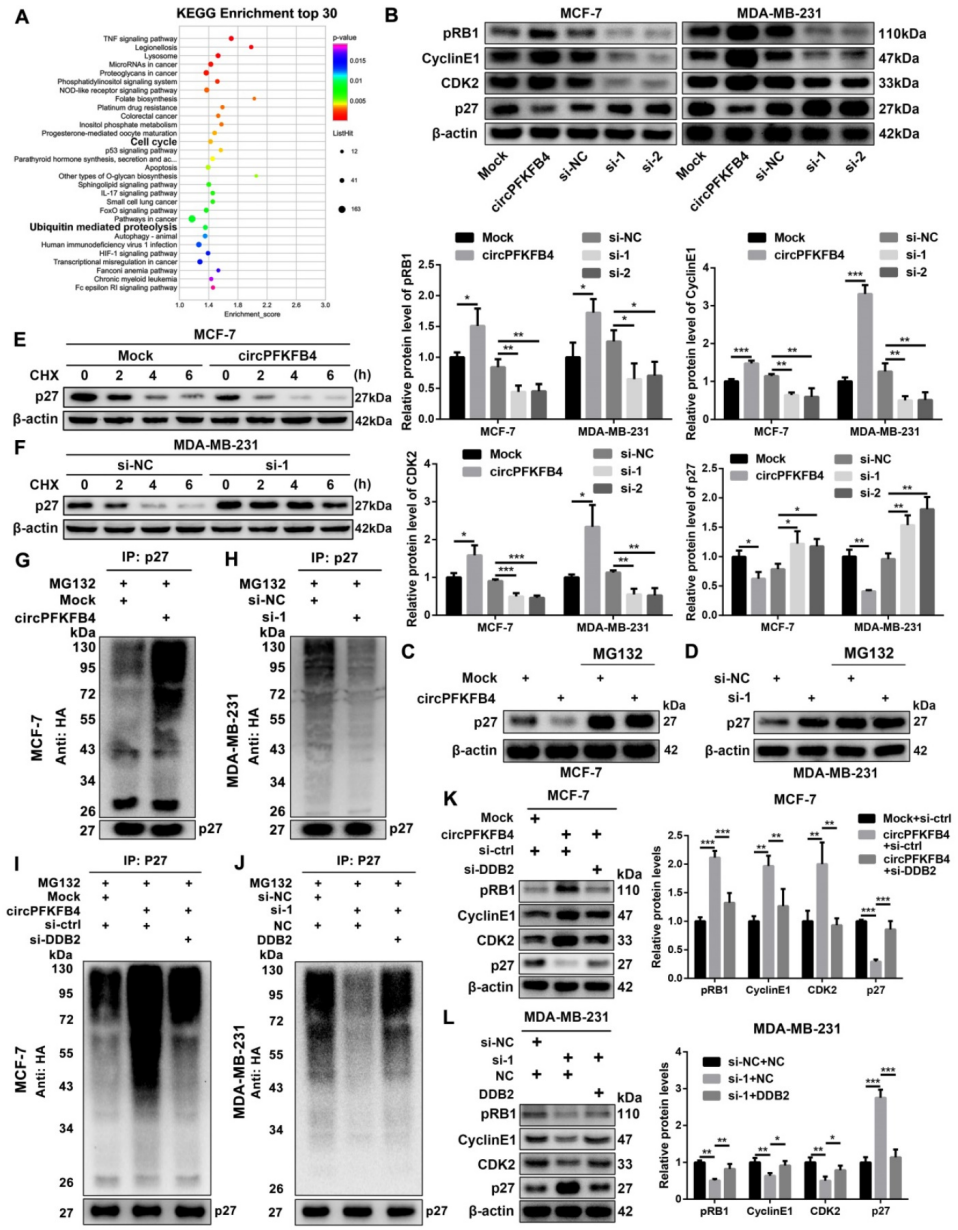
** CircPFKFB4 enhances the ubiquitination and degradation of p27 via DDB2 under hypoxia. (A)** KEGG pathway analysis in hypoxic BC cells after down-regulation of circPFKFB4. **(B)** The protein levels of p27 and its downstream molecules in hypoxic MCF-7 and MDA-MB-231 cells were monitored using western blot. **(C and D)** P27 in circPFKFB4 overexpressed or circPFKFB4 silenced cells cultured under hypoxia with/without 20 µM MG132 treatment for 12 h was detected by western blot. **(E and F)** P27 protein level in hypoxic BC cells transfected with indicated plasmid (E) or siRNAs (F) was measured by western blot after CHX treatment for indicated times. **(G and H)** BC cells were transfected with HA-tagged ubiquitin plasmid, circPFKFB4 overexpression plasmid (G), or siRNAs (H) under hypoxic conditions. At 48 h of post-transfection, the cells were treated with MG132 (20 μM) for 12 h. The cell lysates were immunoprecipitated using anti-p27 antibody, and then the ubiquitylated proteins were checked using anti-HA antibody (top). The cell lysates were immunoblotted with anti-p27 antibody (bottom). **(I and J)** Hypoxic MCF-7 (I) and MDA-MB-231 (J) cells were co-transfected with HA-tagged ubiquitin plasmid and various plasmids as indicated. 48 h after transfection, the cells were harvested and lysed after 12 h of 20 µM MG132 treatment. The samples immunoprecipitated by anti-p27 antibody were immunoblotted with anti-HA antibody (top). The cell lysates were validated by immunoblotting with anti-p27 antibody (bottom). **(K and L)** Hypoxic MCF-7 (K) and MDA-MB-231 (L) cell lysates were analyzed by immunoblotting with the indicated antibodies. Data are presented as mean ± SD and representative of three independent experiments in (B, K and L). *P<0.05, **P<0.01, ***P<0.001.

To further clarify the underlying mechanism of circPFKFB4 in the progression of hypoxic BC cells, we analyzed microarray data in hypoxic MCF-7 cells with circPFKFB4 knockdown. Kyoto Encyclopedia of Genes and Genomes (KEGG) pathway analysis revealed that circPFKFB4 was closely related to ubiquitin-mediated proteolysis and cell cycle signaling pathways under hypoxic conditions (Fig. 7A). Remarkably, a study exploring DDB2 and p27 has reported that DDB2 acts as the substrate specificity receptor of the Cul4A^DDB2^ E3 ubiquitin ligase to degrade p27 22. P27 is acknowledged as a tumor suppressor in a variety of human malignancies 23. Thus, we measured the effect of circPFKFB4 on the protein levels of p27 and its downstream molecules, and found that circPFKFB4 influenced the protein levels of p27 and its downstream molecules, but did not regulate the transcriptional level of p27 under hypoxia (Fig. 7B, Fig. S7E and Fig. S7F). Co-IP experiment between DDB2 and p27 demonstrated that the direct association between DDB2 and p27 was significantly elevated in hypoxic BC cells transfected with circPFKFB4 overexpression plasmid, while the interaction was remarkably impeded following circPFKFB4 knockdown, implying that circPFKFB4 increased the recognition and combination of p27 by the CRL4^DDB2^ ubiquitin ligase (Fig. S8A and B). Additionally, circPFKFB4-mediated p27 downregulation was abolished by MG132 treatment under hypoxic conditions (Fig. 7C and D). Meanwhile, CHX chase assay suggested that the changes in circPFKFB4 expression dramatically affected the half-life of p27 protein in hypoxic BC cells (Fig. 7E and F). The ubiquitin modification level of p27 was increased in circPFKFB4-overexpressing cells but decreased in circPFKFB4-knockdown cells under hypoxia (Fig. 7G and H). Intriguingly, the effect of circPFKFB4 overexpression or silencing on the ubiquitin signals of p27 was restored by DDB2 silencing or overexpression, respectively (Fig. 7I, Fig. 7J and Fig. S9A). Further investigations revealed that depletion of DDB2 reversed the effect of ectopic expression of circPFKFB4 on the levels of p27 and its downstream molecules, whereas the role of circPFKFB4 knockdown in the levels of these molecules was restored by up-regulation of DDB2 under hypoxic conditions (Fig. 7K and L). Together, these data demonstrated that circPFKFB4 promoted the ubiquitination and degradation of p27 by the CRL4^DDB2^ ubiquitin ligase under hypoxia.

### CircPFKFB4 promotes BC progression via DDB2 *in vitro* and *in vivo*

**Figure 8:**
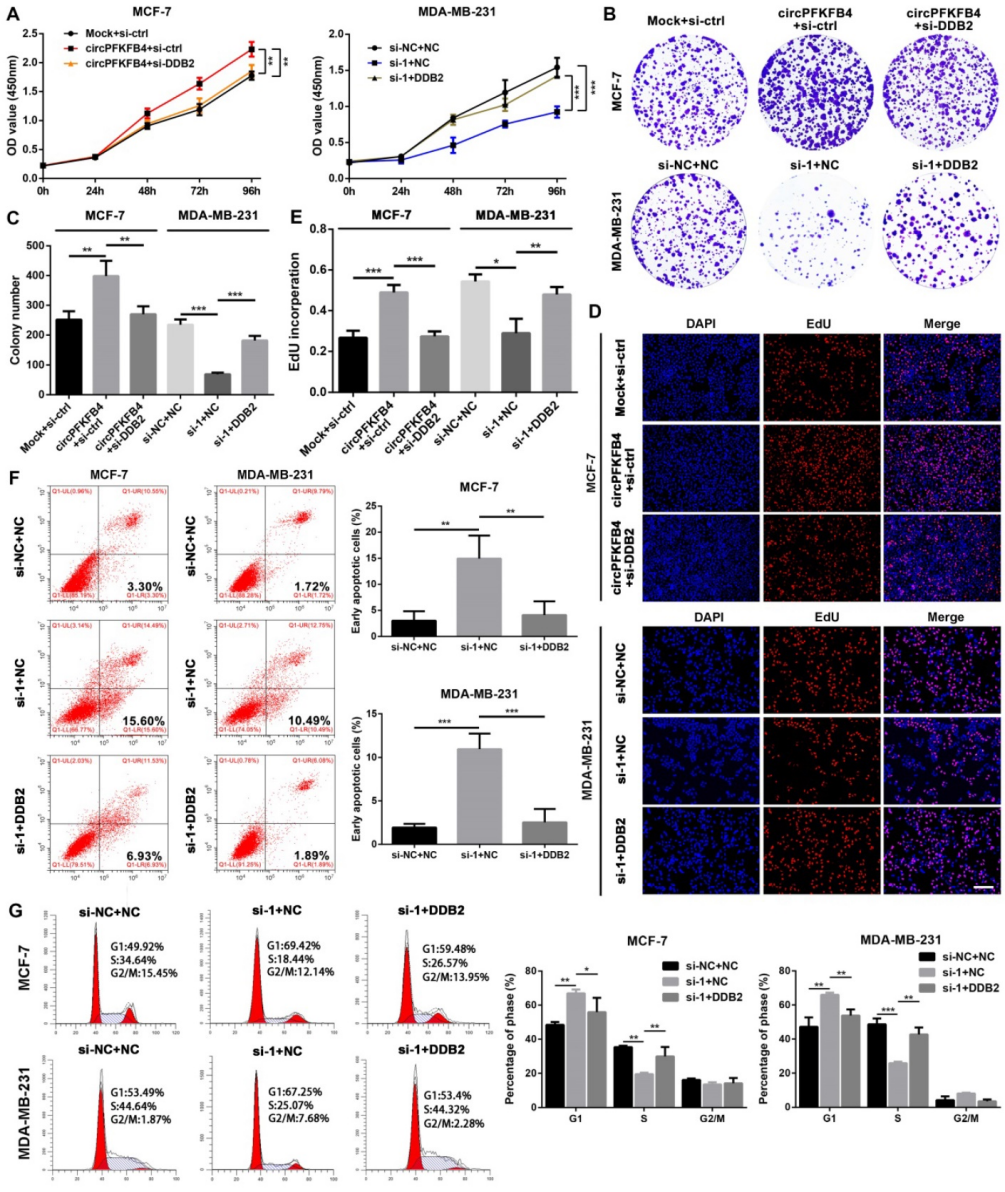
** CircPFKFB4 elevates hypoxic BC cells proliferation *in vitro* by targeting DDB2. (A-E)** CCK-8 (A), colony formation (B and C), and EdU (D and E, scale bar, 200 µm) assays were performed to value the proliferation of BC cells co-transfected with different plasmids and siRNAs as indicated under hypoxia. **(F and G)** Apoptosis rate (F) and cell cycle (G) of hypoxic MCF-7 and MDA-MB-231 cells were calculated using flow cytometry. Data are presented as mean ± SD and representative of three independent experiments in (A-G). *P<0.05, **P<0.01, ***P<0.001.

**Figure 9:**
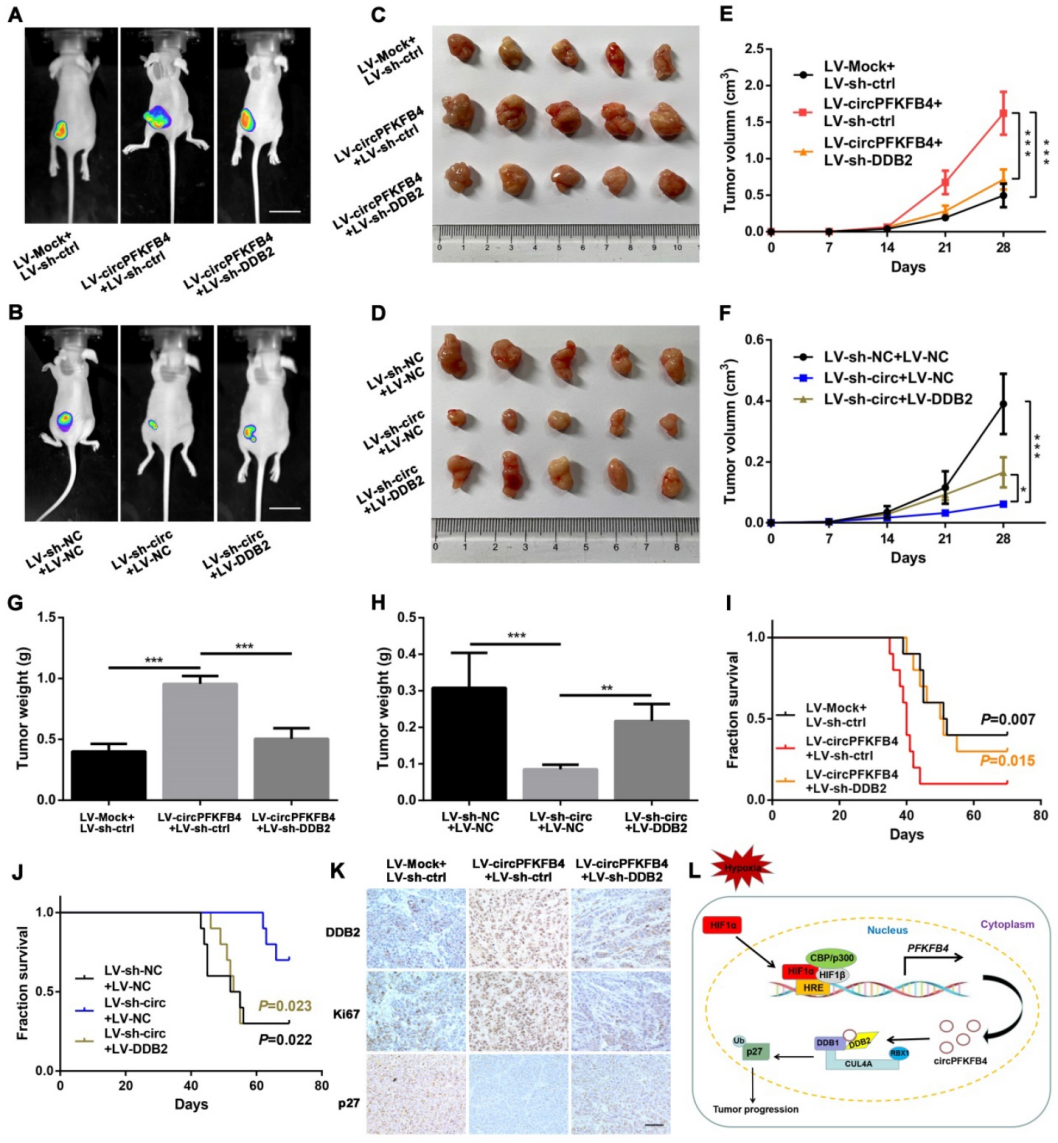
** CircPFKFB4 promotes BC cells growth *in vivo* by targeting DDB2. (A-D)** Representative images of bioluminescence (A and B, scale bar, 2 cm) and xenograft tumors (C and D, n = 5) from each group. **(E and F)** The tumor growth curves were minored (n = 5). **(G and H)** Tumor weight of mice in each group was measured (n = 5). **(I and J)** Overall survival of nude mice in each group (n = 10) was assessed by Kaplan-Meier analysis. **(K)** IHC analyses of DDB2, Ki67, and p27 in xenograft tumors. Scale bar, 100 µm. **(L)** Schematic model displayed that HIF1α-mediated circPFKFB4 promoted the progression of BC through the CRL4^DDB2^ ubiquitin ligase under hypoxia. Data are presented as mean ± SD. *P<0.05, **P<0.01, ***P<0.001.

To further evaluate whether circPFKFB4 exerts its biological roles via DDB2, we completed a series of rescue experiments *in vitro* and *in vivo*. The results of CCK-8, colony formation, and EdU assays suggested that under hypoxic conditions, DDB2 silencing reversed the promoting effect of circPFKFB4 overexpression on BC cell proliferation, while DDB2 overexpression abrogated the inhibitory role of circPFKFB4 knockdown in BC cell proliferation (Fig. 8A-E and Fig. S10A-C). Moreover, the increase of apoptosis induced by depletion of circPFKFB4 could be effectively restored by DDB2 overexpression under hypoxia (Fig. 8F). Furthermore, the cell cycle analysis of hypoxic BC cells suggested that DDB2 overexpression dramatically neutralized cell cycle arrest induced by circPFKFB4 knockdown (Fig. 8G). We further validated these findings by conducting the rescue experiments *in vivo*. The results revealed that LV-circPFKFB4-induced tumor growth was restored by LV-sh-DDB2, vice versa, the inhibitory effect of LV-sh-circ on the tumor growth was weakened by LV-DDB2 (Fig. 9A-H and Fig. S11A). Kaplan-Meier analysis showed that LV-sh-DDB2 or LV-DDB2 abolished the inhibitory or promoting effects of LV-circPFKFB4 or LV-sh-circ on the survival time of nude mice, respectively (Fig. 9I and J). Likewise, IHC indicated that the expressions of DDB2, Ki67, and p27 after overexpression or depletion of circPFKFB4 were remedied by down-regulation or up-regulation of DDB2, respectively (Fig. 9K and Fig. S11B). In summary, these results supported the hypothesis that hypoxia-induced circPFKFB4 promoted the progression of BC by interacting with the CRL4^DDB2^ ubiquitin ligase under hypoxia (Fig. 9L).

## Discussion

Hypoxia is a typical event in solid tumors and the presence of hypoxia has been confirmed in BC 24. Intratumoral hypoxia is a powerful driving force of progression, resistance to chemoradiotherapy and immune suppression of BC, thus leading to the poor survival of BC patients 25. CircRNAs are a class of single-stranded noncoding RNA molecules and have been regarded as potential therapeutic targets in BC 11. However, the roles and molecular mechanisms of circRNAs in BC under hypoxia remain poorly understood. In the present study, the differentially expressed circRNAs in MCF-7 cells with or without hypoxic treatment were screened out using the microarray. We identified a novel circRNA circPFKFB4, which originated from the exon-2, exon-3, and exon-4 of PFKFB4 gene. In clinical investigation, circPFKFB4 was significantly up-regulated in BC tissue samples (81 were estrogen receptor (ER)-positive BC and 19 were triple-negative BC) and closely connected with the adverse clinical stage and poor prognosis of BC patients. Functionally, circPFKFB4 enhanced the growth of ER-positive MCF-7 and triple-negative MDA-MB-231 cells *in vivo* and *in vitro*. Mechanistically, circPFKFB4 promoted the ubiquitination and degradation of p27 by regulating the CRL4^DDB2^ ubiquitin ligase and thereby expediting BC progression.

Recent reports have indicated that circRNAs are implicated in the onset and progression of various cancers under hypoxic conditions. For instance, hypoxia-elevated circRNF20 acts as a miR-487a sponge to upregulate the transcription and protein levels of HIF1α, thus promoting the glycolysis and tumorigenesis of BC 26. Under hypoxia, circ_0008450 facilitates hepatocellular cancer progression by targeting the miR-431/A-kinase anchor protein 1 (AKAP1) axis 27. Hypoxia-induced ebv-circLMP2A up-regulates HIF1α by interacting with KH-type splicing regulatory protein (KHSRP), leading to angiogenesis of Epstein-Barr virus-associated gastric carcinoma 28. Meanwhile, these findings indicated that hypoxia-regulated circRNAs exert their functions by serving as miRNA sponges to regulate their targets. However, the interactions between hypoxia-induced circRNAs and CRLs under hypoxia have not been investigated. We discovered that HIF1α increased the transcription level of circPFKFB4 by directly binding to the PFKFB4 promoter under hypoxia. Moreover, hypoxia-induced circPFKFB4 increased the ubiquitylation and degradation of p27 through an E3 ubiquitin ligase, thereby promoting BC progression. Our data may provide novel insights into the regulatory mechanisms of hypoxia-induced circRNAs in BC development under hypoxia.

RNA pull-down assay combined with mass spectrometry analysis demonstrated that circPFKFB4 could physically bind to both DDB1 and DDB2 under hypoxia. Furthermore, the CRL4^DDB2^ E3 ubiquitin ligase is a vital member of the CRLs family, and its adaptor and substrate receptor proteins are DDB1 and DDB2, respectively 29. These results suggested that circPFKFB4 could interact with the CRL4^DDB2^E3 ubiquitin ligase in hypoxic BC cells. It has been reported that DDB1, one of the two subunits of DNA damage-binding protein complex, is composed of three WD40 β-propeller domains (BPA, BPB, and BPC) and one C-terminal helical domain 29. DDB1 acts as a connector to recruit WD40 repeat proteins (COP1, CSA, and DDB2) containing the DDB1-binding and WD40 repeat (DWD) box to cul4-ROC1 ubiquitin ligase 30. DDB2, a multifunctional WD40 repeat protein, can modulate DNA repair and cancer development through numerous mechanisms. DDB2 consists of an N-terminal helix-loophelix (HLH) domain and a 7-bladed WD40 propeller (WD40) domain. DDB2 identifies and binds to specific DNA/proteins through the WD40 domain 31. Of note, the CRL4^DDB2^ complex can lead to autoubiquitination of DDB2 by targeting the lysine residues at the HLH domain of DDB2 32. The results of domain mapping assay showed that circPFKFB4 combined with BPA and BPC domains of DDB1, as well as the HLH domain of DDB2. CHX chase and ubiquitination experiments demonstrated that the combination of circPFKFB4 and DDB2 might suppress DDB2 autoubiquitination, thus stabilizing the protein level of DDB2 under hypoxia.

The heterodimeric complex of DDB1 and DDB2 further forms the CRL4^DDB2^ ubiquitin ligase complex with CUL4A and RBX1 18. Remarkably, many reports have demonstrated that some proteins can influence the relationship between these components to regulate the assembly of the CRL4^DDB2^ ubiquitin ligase. For instance, UV radiation resistance-associated gene (UVRAG) protein can influence melanoma progression by promoting the formation and activity of the CRL4^DDB2^ E3 ubiquitin ligase through interacting with DDB1 33. The COP9 signalosome (CSN) complex maintains the activity and specificity of the ubiquitin E3 ligase, thus protecting the CRL4^DDB2^ E3 complex from futile auto-ubiquitination 34. Additionally, other studies have indicated that the combination of DDB2 and DDB1 can be disturbed by nuclear receptor interaction protein (NRIP) and PKM2, which disturbs the CRL4^DDB2^ E3 ubiquitin ligase assembly 35, 36. Furthermore, Li et al. first revealed that circACC1 participates in the regulation of the assembly, stability, and activity of AMP-activated protein kinase (AMPK) holoenzyme by directly binding to β and γ subunits of AMPK during metabolic reprogramming. We demonstrated that circPFKFB4 could increase the DDB1-DDB2 interaction, thus enhancing the assembly of the CRL4^DDB2^ ubiquitin ligase complex under hypoxic conditions. Briefly, our experimental results supported the previous findings.

The targets of the CRL4^DDB2^ ubiquitin ligase have been identified, including CDT2, androgen receptor (AR), p27, and PAQR3 22, 37-39. The CRL4^DDB2^ ubiquitin ligase degrades CDT2 to retard colon cancer initiation. The CRL4^DDB2^ ubiquitin ligase inhibits the growth of prostate cancer cells by inducing AR polyubiquitination. The CRL4^DDB2^ ubiquitin ligase influences the ubiquitination level of p27 and regulates cell cycle progression, thereby enhancing the growth of mammalian cells. The CRL4^DDB2^ ubiquitin ligase can also promote the growth and migration of gastric cancer cells by mediating the proteosome degradation of PAQR3. These studies indicated that the CRL4^DDB2^ ubiquitin ligase plays a complicated role in tumorigenesis and progression, which depending on different circumstances and substrates. KEGG pathway analysis indicated that circPFKFB4 was closely related to the cell cycle signaling pathway in hypoxic BC cells. Furthermore, p27 is a cyclin-dependent kinase inhibitor that serves as a key gatekeeper of the G1-to-S phase transition in the cell cycle, which is recognized and ubiquitinated by several E3 ligases 40. Therefore, we focused on the interaction between the CRL4^DDB2^ ubiquitin ligase and p27. Consistent with the previous studies, our results revealed that the CRL4^DDB2^ ubiquitin ligase could ubiquitinate and destabilize p27, and the CRL4^DDB2^ ubiquitin ligase-mediated p27 ubiquitination was increased by circPFKFB4 under hypoxic conditions. P27 is responsible for the resting state of cells by binding to the cyclin E-CDK2 or cyclin D-CDK4 complexes and inhibiting their activation 41. Moreover, as an effective tumor suppressor, p27 is widely involved in the regulation of tumors by non-coding RNAs. For instance, circ_0001326 regulates cell viability by affecting the expressions of p27, cyclin E1, and CDK2 42. Circ BCRC-3 acts as a sponge for miR-182-5p to up-regulate p27 expression, resulting in the suppression of bladder cancer cell proliferation 43. These findings are consistent with our results that circPFKFB4 decreased the protein expression of p27 but increased the protein levels of its downstream molecules including cyclin E1, CDK2, and pRB1, suggesting that circPFKFB4 stimulated BC cell proliferation via the p27 signaling pathway under hypoxia.

## Conclusion

In conclusion, our study demonstrates for the first time that circPFKFB4, whose transcription is promoted by HIF1α under hypoxia, is up-regulated in BC and associated with poor prognosis. CircPFKFB4 facilitates the binding of DDB1 and DDB2 and promotes the degradation of p27 mediated by the CRL4^DDB2^ ubiquitin ligase, eventually leading to BC progression under hypoxia. Our results contribute to deepening our knowledge of the detailed functions and intricate molecular mechanisms of circPFKFB4 and providing new insights into potential biomarkers and therapeutic targets for BC.

## Supplementary Material

Supplementary figures and table.

## Figures and Tables

**Table 1:** Correlation between circPFKFB4 expression and clinicopathological parameters in 100 BC patients

Characteristic	Cases	CircPFKFB4		Chi-square	*P* value
		Low	High		
**Age (years)**					
<55	74	38	36	0.208	0.648
≥55	26	12	14		
**Menopausal**					
Premenopausal	47	22	25	0.361	0.548
Postmenopausal	53	28	25		
**TNM stage**					
I-II	84	43	41	0.298	0.585
III	16	7	9		
**Tumor size (cm)**					
≤2	52	31	21	4.006	0.045*
>2	48	19	29		
**N stage**					
0	52	24	28	0.641	0.423
I-III	48	26	22		
**Grade**					
I-II	62	32	30	0.170	0.680
III	38	18	20		

*P<0.05.

**Table 2:** Correlation between circPFKFB4 expression and clinicopathological variables in 280 BC patients

Characteristic	Cases	CircPFKFB4		Chi-square	*P* value
		Low	High		
**Age (years)**					
<55	168	93	75	1.739	0.187
≥55	112	53	59		
**TNM stage**					
I-II	189	108	81	5.826	0.016*
III	91	38	53		
**Tumor size (cm)**					
≤2	90	57	33	6.656	0.010*
>2	190	89	101		
**N stage**					
0	136	76	60	1.482	0.223
I-III	144	70	74		
**Grade**					
I-II	245	132	113	2.364	0.124
III	35	14	21		

*P<0.05.

**Table 3:** Univariate and multivariate Cox regression analysis of circPFKFB4 and survival in 278 BC patients

Clinical variables	Univariate analysis		*P* value	Multivariate analysis		*P* value
	HR	95%CI		HR	95%CI	
Age (< 55 vs. ≥55 years)	1.317	0.857-2.021	0.209			
TNM stage (I-II vs. III)	2.191	1.430-3.357	0.000***	3.439	1.738-6.804	0.000***
Tumor size (≤2 vs. >2 cm)	1.507	0.972-2.449	0.098			
N stage (0 vs. I-III)	1.245	0.811-1.909	0.316			
Grade (I-II vs. III)	1.437	0.796-2.594	0.230			
CircPFKFB4 (low vs. high)	2.007	1.294-3.112	0.002**	1.731	1.109-2.702	0.016*

Abbreviations: HR, Hazard ratio; CI, Confidence interval. *P<0.05, **P<0.01, ***P<0.001.

## Abbreviations

AUC: Area under the curve
BC: Breast cancer
BPA: β-propelle A
BPB: β-propelle B
BPC: β-propelle C
CCK-8: Cell counting kit-8
cDNA: Complimentary DNA
ChIP: Chromatin immunoprecipitation
CHX: Cycloheximide
CircRNA: Circular RNA
Co-IP: Co-immunoprecipitation
CRLs: Cullin-RING ubiquitin ligases
CUL4A: Cullin4A
DDB1: Damage-specific DNA-binding protein 1
DDB2: Damage-specific DNA-binding protein 2
DWD: DDB1-binding and WD40
HR: Hazard ratio
EdU: 5-Ethynyl-20- deoxyuridine
FISH: Fluorescence *in situ* hybridization
HIF1α: Hypoxia-inducible factor 1α
HLH: Helix-loophelix
HREs: Hypoxia response elements
IF: Immunofluorescence
IHC: Immunohistochemistry
IP: Immunoprecipitation
ISH: *In situ* hybridization
KEGG: Kyoto Encyclopedia of Genes and Genomes
qRT-PCR: Quantitative real-time polymerase chain reaction
RBX1: RING-box protein 1
RIP: RNA immunoprecipitation
ROC: Receiver operating characteristic
