# Supplementary material for: Hypoxia-inducible CircPFKFB4 Promotes Breast Cancer Progression by Facilitating the CRL4DDB2 E3 Ubiquitin Ligase-mediated p27 Degradation
Source: Int J Biol Sci. 2022 Jun 6;18(9):3888–907. doi: 10.7150/ijbs.72842 (PMC9254479; doi:10.7150/ijbs.72842)
Supplement: Supplementary file 1 — Supplementary figures and table. [file ijbsv18p3888s1.pdf]

**Supplementary Table S1. Sequences of primers used in this study.**

| Gene | Primer sequences |
|---|---|
| circPFKFB4 (Divergent) | F: 5’-ATCATTCCAATATTCCTCAATAGTG-3’<br>R: 5’-GTCAGCTTCTTGGAGATGTA-3’ |
| circPFKFB4 (Convergent) | F: 5’-GGGCAAGACCTACATCTCCA-3’<br>R: 5’-TTTTCAGGCCCTCTTCATTG-3’ |
| hsa_circ_0121519 | F: 5’-TTAAGATTAGGGAGGAGCAC-3’<br>R: 5’-CAGGCTCCTACTGTATTCTG-3’ |
| hsa_circ_0042496 | F: 5’-GCAAGCCTTGCTGATGATAAT-3’<br>R: 5’-AGCAAGACTCCAGACTATATCTC-3’ |
| hsa_circ_0081572 | F: 5’-ACGTCAGCCATGTACAGGAT-3’<br>R: 5’-GCGTGGTGAACTCAGTGACT-3’ |
| hsa_circ_0042493 | F: 5’-TGGGACCTGAAAGATGATGT -3’<br>R: 5’-GCAAGACTCCAGACTATATCTC-3’ |
| β-actin (Divergent) | F: 5’-AAATCGTGCGTGACATTAAGGAGA-3’<br>R: 5’-CATACCCCTCGTAGATGGGCA-3’ |
| β-actin (Convergent) | F: 5’-CATGTACGTTGCTATCCAGGC-3’<br>R: 5’-CTCCTTAATGTCACGCACGAT-3’ |
| HIF1α | F: 5’-GAACGTCGAAAAGAAAAGTCTCG-3’<br>R: 5’-CCTTATCAAGATGCGAACTCACA -3’ |
| U6 | F: 5’-CTCGCTTCGGCAGCACA-3’<br>R: 5’-AACGCTTCACGAATTTGCGT-3’ |
| DDB2 | F: 5’-CTCCTCAATGGAGGGAACAA-3’<br>R: 5’-GTGACCACCATTCGGCTACT-3’ |
| p27 | F: 5’-TCGGGGTCTGTGTCTTTTGG-3’<br>R: 5’-AGACACTCGCACGTTTGACA-3’ |
| PFKFB4 promoter 1 | F: 5’-TAAGATACTCCCAGGGCTTAGTG-3’<br>R: 5’-TTTAGTAGAGGCGGGATTTCACC-3’ |
| PFKFB4 promoter 2 | F: 5’-TCCCGCCTCTACTAAAAATACAA-3’<br>R: 5’-TGCTGGGAGCAAATGCGTGGGAC-3’ |
| PFKFB4 promoter 3 | F: 5’-AGCAGCCAAAGCAAAGAAAG-3’<br>R: 5’-ACTCTTCCGCCGTCCATAG-3’ |
| PFKFB4 promoter 4 | F: 5’-TCCCTAGCAAGGAGGTAGCA-3’<br>R: 5’-GCAAACTCAGCTCTCCCAAC-3’ |
| PFKFB4 promoter 5 | F: 5’-GATCCTGGCCTGAAGAACTG-3’<br>R: 5’-GCGACAGCCCATGTCTATCT-3’ |

**Supplementary Figure S1**

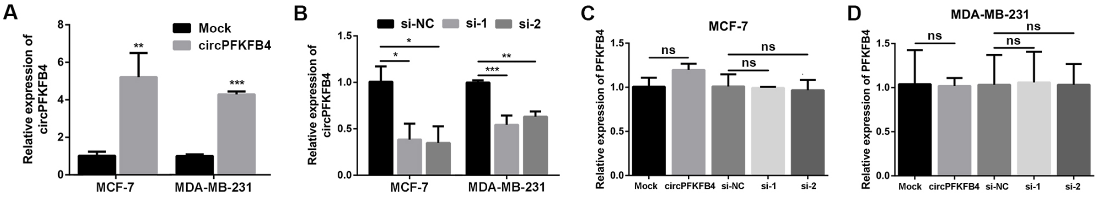


**Supplementary Figure S1. The expression levels of circPFKFB4 and PFKFB4 in BC cells under hypoxia.** (A-D) The relative expressions of circPFKFB4 (A and B) and PFKFB4 (C and D) were detected by qRT-PCR in BC cells after circPFKFB4 up-regulation (circPFKFB4) or down-regulation (si-1 and si-2) under hypoxia. Data are presented as mean ± SD and representative of three independent experiments in (A-D). **P*<0.05, ***P*<0.01, ****P*<0.001, ns, no significance.

**Supplementary Figure S2**

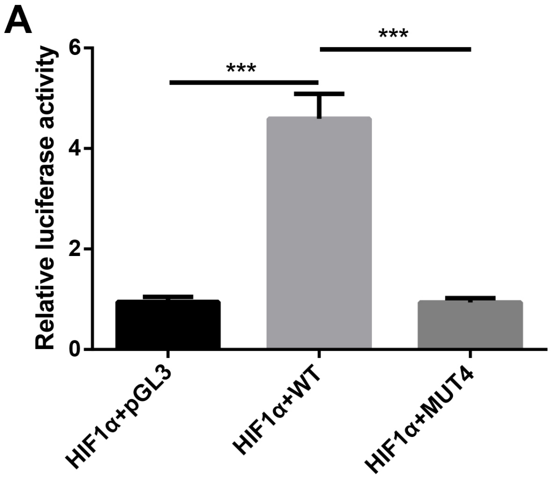


**Supplementary Figure S2. HIF1α binds directly to the PFKFB4 promoter under hypoxia.** (A) Hypoxic MCF-7 cells were transfected with pGL3, WT, or MUT4 along with HIF1α, and the luciferase activity was determined using luciferase activity assay. Data are presented as mean ± SD and representative of three independent experiments in (A). ****P*<0.001.

## Supplementary Figure S3

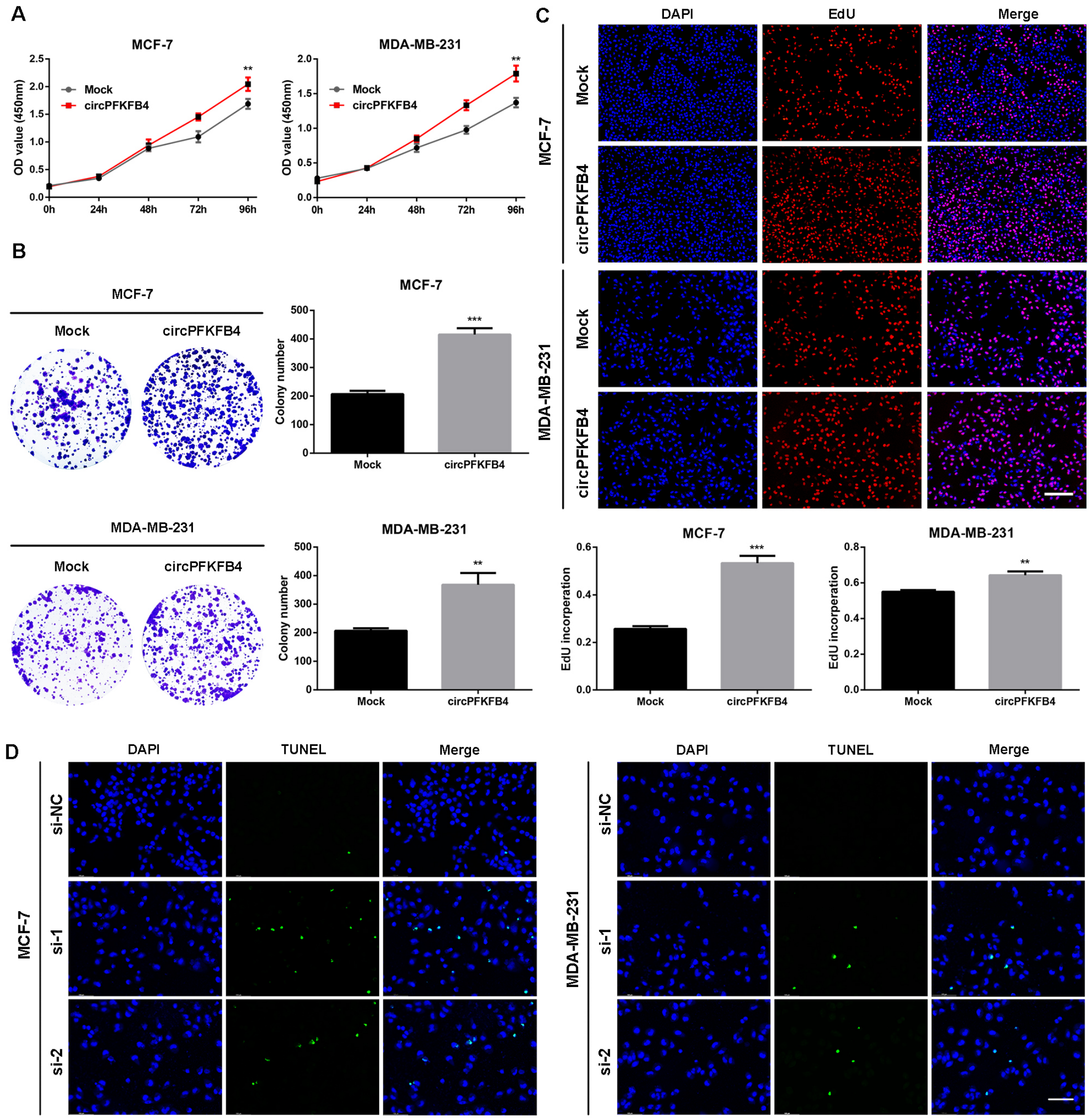


**Supplementary Figure S3. CircPFKFB4 facilitates the proliferation and reduces apoptosis of BC cells under hypoxia.** (A-C) The proliferation of hypoxic BC cells transfected with the ectopic plasmid of circPFKFB4 was measured using CCK-8 (A), colony formation (B), and EdU (C, scale bar, 200 μm) assays. (D) Apoptosis in hypoxic BC cells was detected using TUNEL. Scale bar, 100 μm. Data are presented as mean ± SD and representative of three independent experiments in (A-C). **P*<0.05, ***P*<0.01, ****P*<0.001.

**Supplementary Figure S4**

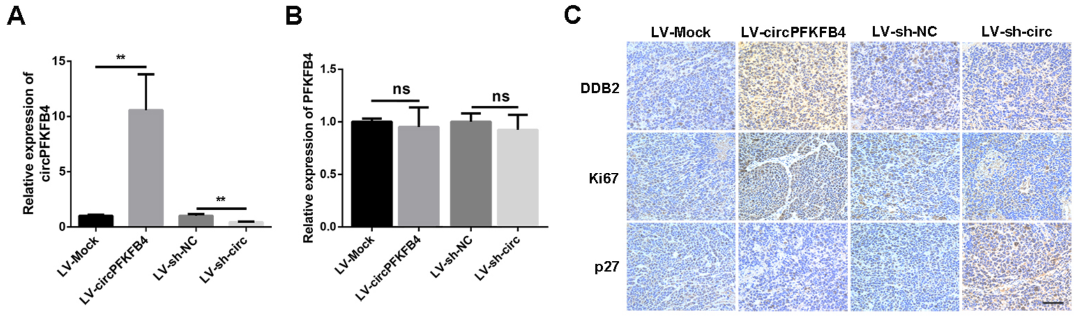


**Supplementary Figure S4. The expression levels of DDB2, Ki-67 and p27 in xenograft tumors.** (A and B) qRT-PCR was used to detect circPFKFB4 (A) and PFKFB4 (B) expressions in MCF-7 cells infected with the indicated lentivirus under hypoxic conditions. (C) IHC of DDB2, Ki-67, and p27 in xenograft tumors. Scale bar, 100 μm. Data are presented as mean ± SD and representative of three independent experiments in (A and B). **P<0.01, ns, no significance.

## Supplementary Figure S5

A

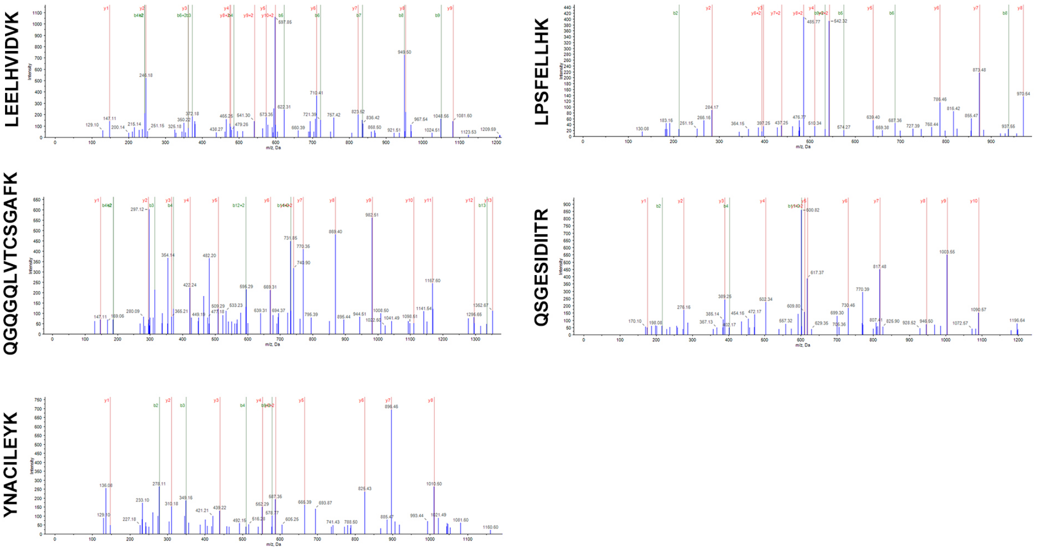


**Supplementary Figure S5. circPFKFB4 directly binds to DDB1 under hypoxia.**

(A) Unique peptides of DDB1 identified by mass spectrometry analysis.

# Supplementary Figure S6

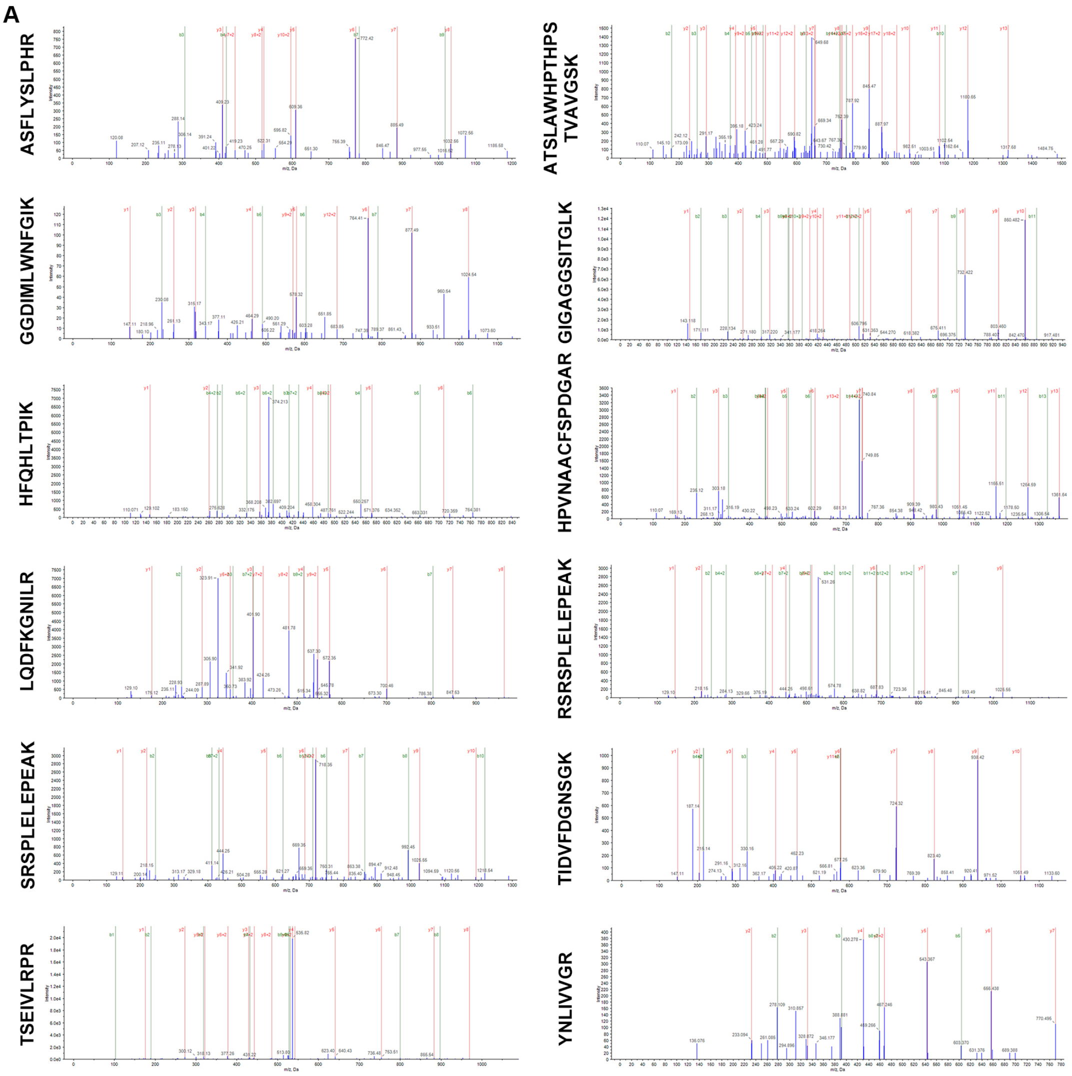


**Supplementary Figure S6. circPFKFB4 directly binds to DDB2 under hypoxia.**

(A) Unique peptides of DDB2 identified by mass spectrometry analysis.

## Supplementary Figure S7

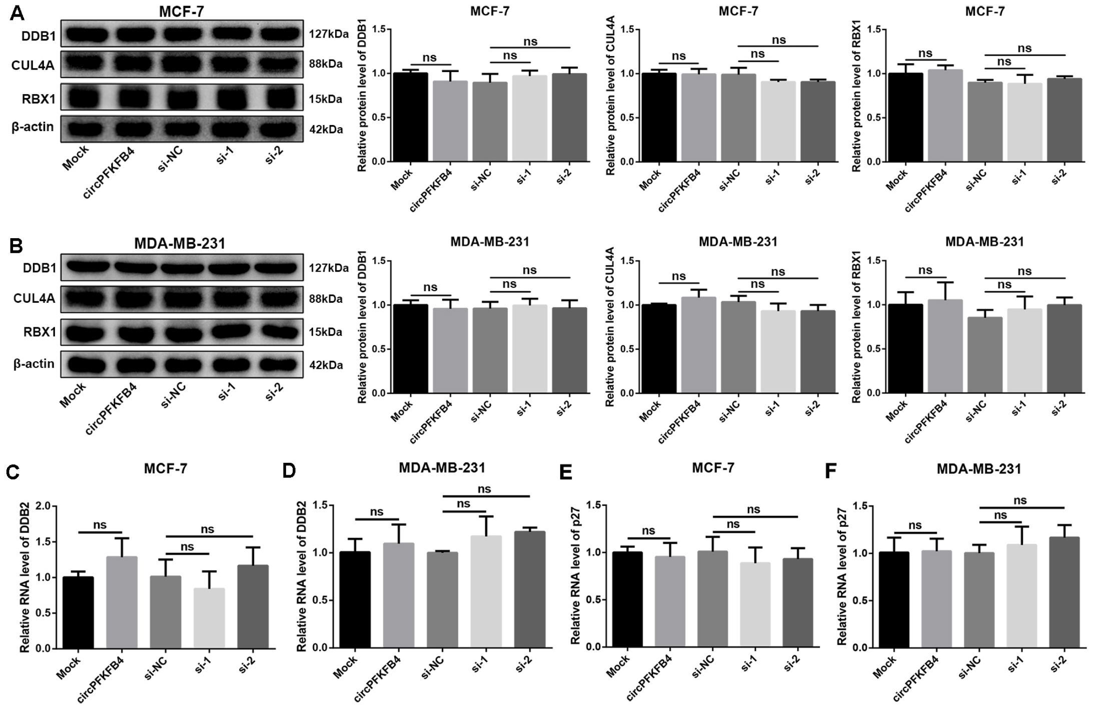


**Supplementary Figure S7. CircPFKFB4 does not affect the protein levels of DDB1, CUL4A, and RBX1, and does not regulate the mRNA levels of DDB2 and p27.** (A and B) The effect of up-regulation or down-regulation of circPFKFB4 on the expressions of DDB1, CUL4A, and RBX1 in hypoxic MCF-7 (A) and MDA-MB-231 (B) cells was measured using Western blot. (C-F) The effect of circPFKFB4 on the mRNA levels of DDB2 (C and D) and p27 (E and F) in hypoxic MCF-7 and MDA-MB-231 cells was detected using qRT-PCR. Data are presented as mean ± SD and representative of three independent experiments in (A-F). ns, no significance.

**Supplementary Figure S8**

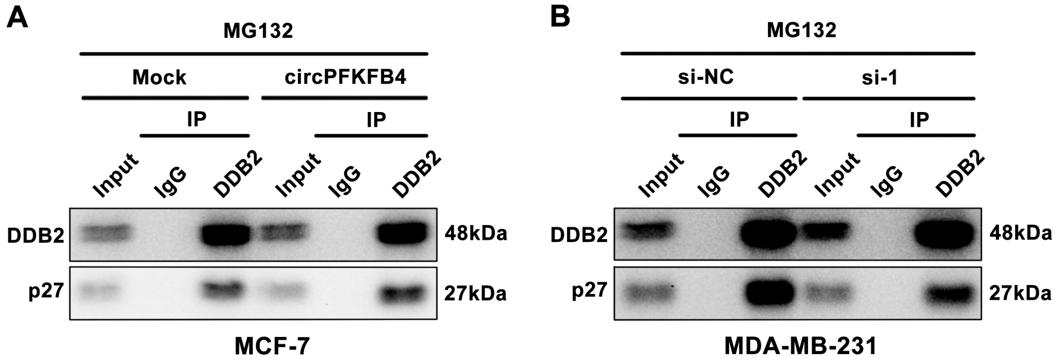


**Supplementary Figure S8. CircPFKFB4 increases the recognition and combination of p27 via DDB2 under hypoxia.** (A and B) Co-IP assay was applied in hypoxic MCF-7 (A) and MDA-MB-231 (B) cells lysates using anti-DDB2, anti-p27, or anti-IgG, and then, the levels of DDB2 and p27 were detected using western blot.

Supplementary Figure S9

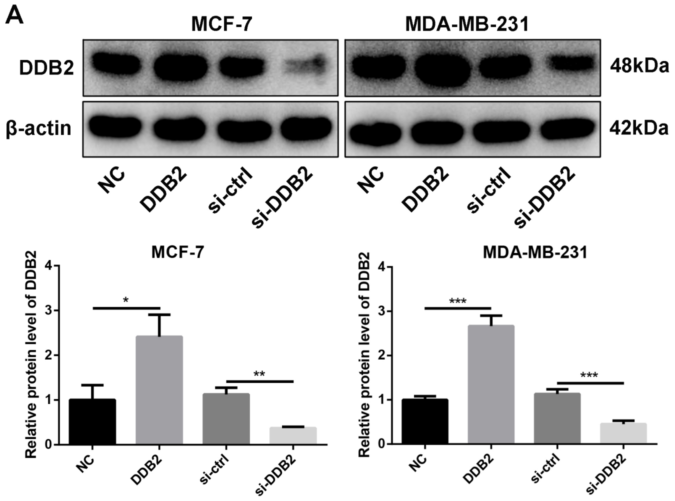


**Supplementary Figure S9. DDB2 protein level in MCF-7 and MDA-MB-231 cells cultured under hypoxia.** (A) The protein level of DDB2 in hypoxic BC cells transfected with DDB2 overexpression plasmid or si-DDB2 was determined using western blot. Data are presented as mean ± SD and representative of three independent experiments in (A). **P*<0.05, ***P*<0.01, ****P*<0.001.

## Supplementary Figure S10

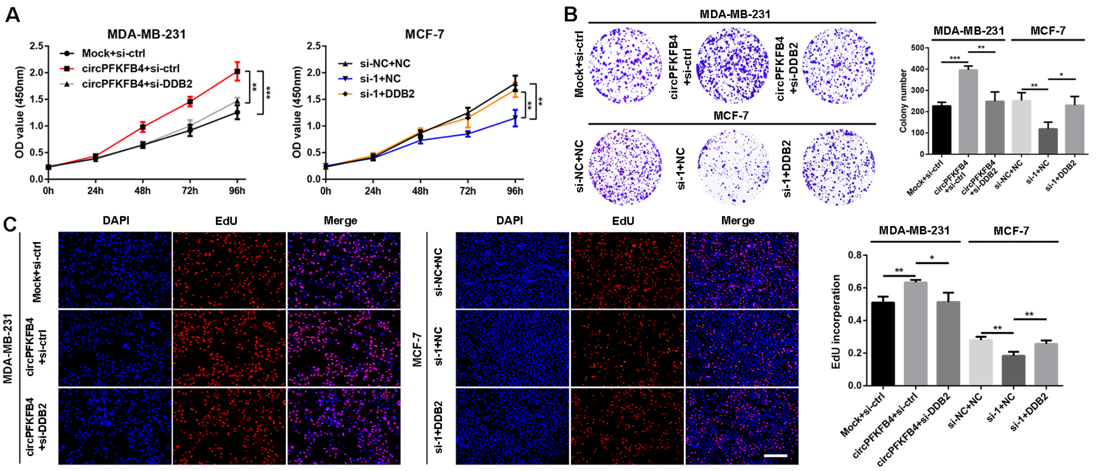


**Supplementary Figure S10. CircPFKFB4 affects hypoxic BC cells proliferation via DDB2.** (A-C) **Hypoxic** BC cells proliferation was evaluated by CCK-8 (A), colony formation (B), and EdU (C, scale bar, 200 μm) assays. Data are presented as mean ± SD and representative of three independent experiments in (A-C). **P*<0.05, ***P*<0.01, ****P*<0.001.

## Supplementary Figure S11

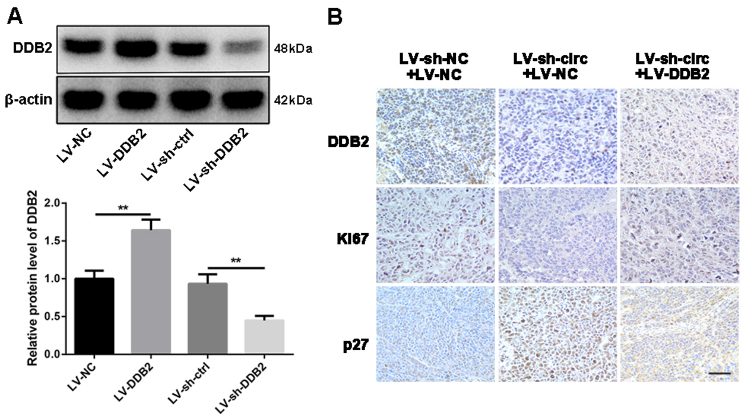


**Supplementary Figure S11. CircPFKFB4 and DDB2 are involved in tumor growth.** (A) Western blot analysis of MCF-7 cells with stably forced or silenced DDB2 expression under hypoxic circumstances. (B) IHC staining of DDB2, Ki67, and p27 in subcutaneous xenograft tumors. Scale bar, 100 μm. Data are presented as mean ± SD and representative of three independent experiments in (A). ***P*<0.01.
